# Deciphering the genetic regulation of peripheral blood transcriptome in pigs through expression genome-wide association study and allele-specific expression analysis

**DOI:** 10.1186/s12864-017-4354-6

**Published:** 2017-12-13

**Authors:** T. Maroilley, G. Lemonnier, J. Lecardonnel, D. Esquerré, Y. Ramayo-Caldas, M. J. Mercat, C. Rogel-Gaillard, J. Estellé

**Affiliations:** 1 GABI, INRA, AgroParisTech, Université Paris-Saclay, 78350 Jouy-en-Josas, France; 2 GenPhySE, INRA, INPT, ENVT, Université de Toulouse, 31326 Castanet-Tolosan, France; 3 IFIP - Institut du porc/BIOPORC, La Motte au Vicomte, BP 35104, 35651 Le Rheu, France

**Keywords:** Blood, Pig, Transcriptome, eQTL, eGWAS, RNA-Seq, Allele-Specific Expression

## Abstract

**Background:**

Efforts to improve sustainability in livestock production systems have focused on two objectives: investigating the genetic control of immune function as it pertains to robustness and disease resistance, and finding predictive markers for use in breeding programs. In this context, the peripheral blood transcriptome represents an important source of biological information about an individual’s health and immunological status, and has been proposed for use as an intermediate phenotype to measure immune capacity. The objective of this work was to study the genetic architecture of variation in gene expression in the blood of healthy young pigs using two approaches: an expression genome-wide association study (eGWAS) and allele-specific expression (ASE) analysis.

**Results:**

The blood transcriptomes of 60-day-old Large White pigs were analyzed by expression microarrays for eGWAS (242 animals) and by RNA-Seq for ASE analysis (38 animals). Using eGWAS, the expression levels of 1901 genes were found to be associated with expression quantitative trait loci (eQTLs). We recovered 2839 local and 1752 distant associations (Single Nucleotide Polymorphism or SNP located less or more than 1 Mb from expression probe, respectively). ASE analyses confirmed the extensive *cis*-regulation of gene transcription in blood, and revealed allelic imbalance in 2286 SNPs, which affected 763 genes. eQTLs and ASE-genes were widely distributed on all chromosomes. By analyzing mutually overlapping eGWAS results, we were able to describe putative regulatory networks, which were further refined using ASE data. At the functional level, genes with genetically controlled expression that were detected by eGWAS and/or ASE analyses were significantly enriched in biological processes related to RNA processing and immune function. Indeed, numerous distant and local regulatory relationships were detected within the major histocompatibility complex region on chromosome 7, revealing ASE for most class I and II genes.

**Conclusions:**

This study represents, to the best of our knowledge, the first genome-wide map of the genetic control of gene expression in porcine peripheral blood. These results represent an interesting resource for the identification of genetic markers and blood biomarkers associated with variations in immunity traits in pigs, as well as any other complex traits for which blood is an appropriate surrogate tissue.

**Electronic supplementary material:**

The online version of this article (10.1186/s12864-017-4354-6) contains supplementary material, which is available to authorized users.

## Background

Modern livestock breeding programs aim to foster sustainability and improve production by selecting animals for health- and environment-related phenotypes. However, complex health traits such as immune capacity, disease resistance, and robustness are difficult to directly measure in living animals. There is a strong need for the identification of intermediate phenotypes that can be used as proxies for these traits. In pigs, data on immune function are regularly obtained by scoring innate and adaptive immunity traits measured in blood and serum (e.g., blood cell counts, specific and non-specific antibodies, and acute phase proteins) or by studying the effect of in vitro stimulation of total blood or leucocytes (e.g., production of chemokines and cytokines, phagocytosis) [1, 2]. We and others have reported that many immunity traits are heritable in swine [1–3]. We have also shown that changes in the peripheral blood transcriptome are correlated to variation in immune traits [4]. In this context, then, peripheral blood appears to be a relevant tissue with which to phenotype immunity traits, as well as a relevant surrogate tissue for the quantification of other physiological traits.

Variation in transcript abundance has been studied as an inherited quantitative trait [5] and association mapping of loci for expression traits has been performed in several species for a wide range of tissues [6]. Indeed, as demonstrated by Schadt et al. [7], characterizing the relationships between DNA sequence and RNA expression is an appropriate step in understanding the links between genotype and phenotype. In the first global analysis of expression quantitative trait loci (eQTLs) conducted in mammals, these authors showed that gene expression traits in mice were connected with complex phenotypes like obesity. eQTLs can control the expression levels of local and/or distant transcripts [8] and are nowadays identified by expression genome-wide association studies (eGWASs). Local and distant associations are often referred to as *cis*- and *trans*-associations and correspond to putative *cis*- and *trans*-regulatory relationships, which act, respectively, in an allele-specific and non-allele-specific manner. Complementarily to gene expression microarrays, an RNA-Seq-based approach provides sequencing data for the detection of both transcript expression levels and single-nucleotide polymorphisms (SNPs). By integrating these two types of data, allele-specific expression (ASE) analyses provide information on the relative expression levels of two alleles of a gene from the same individual, thus targeting *cis-*acting regulation [9]. An approach that combines eGWAS and ASE techniques is highly effective in characterizing candidate genetic variants acting as *cis*- and *trans*-regulators, as has been shown in the mouse for liver [10] and adipose tissue [11].

In humans, results on the genetic control of the blood transcriptome were reported to be robust and reproducible despite possible confounding effects due to variations in white and red cell counts [33]. These results highlighted the utility of blood eQTL mapping for improving the interpretability of GWAS results for complex phenotypes, especially by helping with the prioritization of candidate genes. Similarly, Schramm et al. [31] showed that eQTL analysis of whole blood is reliable and may be used to identify biomarkers and to enhance understanding of the molecular mechanisms underlying associations between genes and disease. Together, all these eQTL data have provided new types of resources and associated databases for the study of relationships between gene expression and phenotype of interest [12].

Few eGWASs have been reported in pigs. Liaubet et al. [13] analyzed gene expression in pig skeletal muscle sampled shortly after slaughtering, and showed an over-representation of genes that encoded proteins involved in processes induced during muscle development and metabolism, cell morphology, stress response, and apoptosis. More recently, eGWASs have been integrated with phenotyping studies in order to identify candidate genes and causative mutations associated with phenotypic variations in several tissues: for example, liver gene expression linked to blood and lipid traits [41], gene expression in *longissimus dorsi* or *gluteus medius* muscles associated with growth, fatness, yield, and meat quality [14–19], and hypothalamus gene expression connected to coping behavior [20].

To our knowledge, no eGWAS of total blood has yet been performed in pigs. In this study, our aim was to build a genome-wide map of the genetic control of gene expression in the blood of 60-day-old Large White pigs by combining eGWAS and ASE results. Such a resource will be valuable for guiding integrated studies of complex phenotypes, as has been demonstrated in studies of humans [12], especially those linked to health and immune response in young pigs, which are prone to be subjected to infection challenges. After detecting the eQTL and ASE effects, we performed*in silico* functional analyses in order to illustrate the usefulness of our data in not only prioritizing candidate blood biomarkers and SNPs associated with immunity and physiological trait variations but also in deciphering the complexity of blood gene transcriptional regulation in pigs.

## Results

### eGWAS revealed numerous cases of local and distant regulation of gene transcription in blood

We performed an eGWAS by combining expression data from customized single-channel 8X60K Agilent arrays and SNP genotypes from Illumina PorcineSNP60K genotyping chips taken from 242 French Large White pigs. We searched for significant associations between all expressed probes that mapped to a unique position on the Sscrofa10.2 reference genome (39,649 probes from a total of 59,774 expressed probes) and all SNPs on the reference genome that met the quality control threshold (42,234 SNPs from a total of 61,557). For these association analyses, all probes, including those annotated to the same gene, were considered independent.

**Fig. 1:**
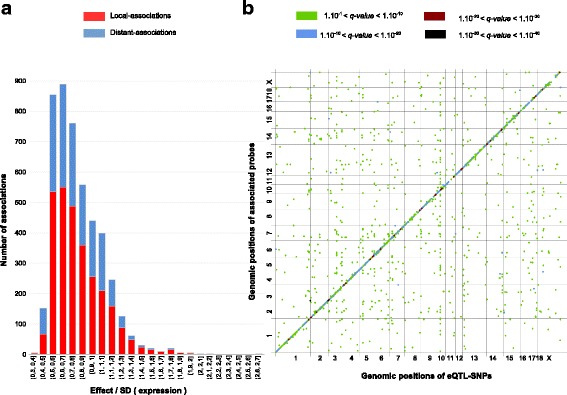
Overview of associations identified by eGWAS. **a**: The distribution of additive effects of local-acting (red bars) or distant-acting (blue bars) eQTL-SNPs. eQTL-SNP effects are expressed as the ratio between the effect of the eQTL-SNP on probe expression and the standard deviation of variation in probe expression. **b**: Genomic locations of expressed probes versus genomic positions of associated eQTL-SNPs. Each significant association is represented by a dot; the color of dots indicates the significance (q-value) of the association (green: 1x10^−1^ < *q* < 1x10^−10^, dark red: 1x10^−10^ < *q* < 1x10^−20^, blue: 1x10^−20^ < *q* < 1x10^−30^, black: 1x10^−30^ < *q* < 1x10^−40^). Dots on the diagonal represent local associations; off-diagonal dots correspond to distant associations

Overall, we identified 4591 associations, which involved 3195 eQTL-SNPs (see below) and 3419 probes that were retained for further analysis (Fig. 1 and Additional file 1: Table S1). Initially, 48,536 significant associations (FDR < 0.05) were detected between 18,541 SNPs and 3419 expressed probes mapping to an unique genomic position. In order to reduce redundancy in SNPs that was due to linkage disequilibrium, we represented the signals of all associated SNPs that harbored the same effect on probe expression by the most significant SNP in a window of 5 Mb. We used this SNP to represent the eQTL in further analyses and designated it as an eQTL-SNP. From this subset of eQTL-SNPs, 77% of the 3419 associated probes could be assigned to 1901 pig genes. Probe annotation revealed that gene assignment included alignments of sense (1828 genes), antisense (73 genes), or both sense and antisense (244 genes) probe sequences (Additional file 2: Table S2). In addition, 421 probes could not be assigned to any known gene but were found associated with at least one eQTL-SNP.

A large majority of eQTL-SNPs (*N* = 2454) was associated with variation in the expression of a single probe. The other 965 eQTL-SNPs were each associated with between two (483 eQTL-SNPs) and 51 probes (one eQTL-SNP) (Table 1). Reciprocally, probes were associated with up to six eQTL-SNPs, although most were linked to only one or two (Table 1).

**Table 1:** Numbers of significant associations between probes and eQTL-SNPs, and vice versa

	Number of associated eQTL-SNPs or probes											
	1	2	3	4	5	6	7	8	9	10	11–51	Total
Probes	2423	839	141	14	1	1						3419
eQTL-SNPs	2454	483	146	44	23	16	7	7	2	4	9	3195

Among all associations, we characterized 2839 as local and 1752 as distant. In local associations, candidate eQTL-SNPs were located within 1 Mb of the start position of the associated probe and were expected to act on that probe’s expression in an allele-specific manner, meaning a *cis*-regulatory action. All other associations were classified as distant associations and the corresponding eQTL-SNPs are referred to as distant eQTL-SNPs, expected to act in *trans* by regulating the associated gene in a non-allele-specific manner. Among the 3195 eQTL-SNPs, 2124 were local and 1187 were distant, while 116 were involved in both local and distant associations. Furthermore, 2407 probes were affected by at least one local eQTL-SNP and 1427 probes by at least one distant eQTL-SNP, with 415 probes associated with at least one local and one distant eQTL-SNP. For 2382 local associations (> 83%), the eQTL-SNP was located less than 500 kb from the start position of the probe, and for a subset of 1334 associations, the eQTL-SNP was within 100 kb of the associated probe.

In order to evaluate the degree of the eQTL-SNP effect with respect to global variation in probe expression, we calculated the ratio of the eQTL-SNP effect to the standard deviation of the expression of its associated probe. The ratio distribution is represented in Fig. 1a and showed that eQTL-SNPs had a wide range of additive effect sizes on the expression variation of associated probes. The means of local and distant eQTL-SNP effects were significantly different (unilateral Student test <0.05), with local associations showing larger effects. Globally, the median ratio was equal to 0.8, meaning that for a majority of associations, the effect of the eQTL-SNP was nearly equal to the standard deviation of the gene probe expression. For 930 associations, the effect of the eQTL-SNP was larger than the standard deviation (ratio higher than 1; Fig. 1a). In eight extreme cases, the effect was at least two times higher than the standard deviation of gene expression. The most extreme association had a ratio of 2.68; this involved the *IFITM2* gene on SSC2 and was also the most significant association found by eGWAS (q-value = 1.25 × 10^−39^).

**Fig. 2:**
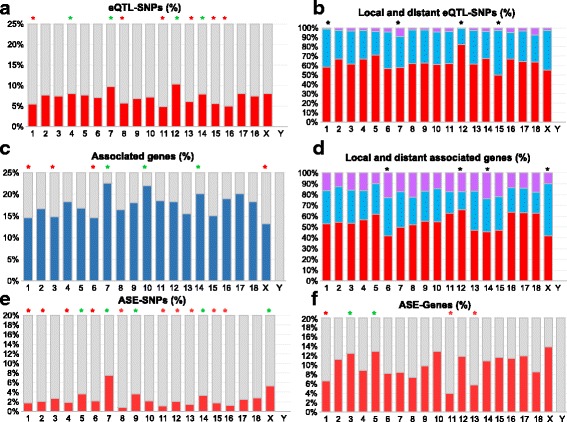
Genome-wide distribution of associations identified by eGWAS (A-D), and ASE-SNPs or -genes (E-F). **a**: Per-chromosome percentage of eQTL-SNPs detected by eGWAS (red) in the larger SNP set. **b**: Relative proportions of SNPs that were detected as local-acting (red), distant-acting (blue) or both local- and distant-acting (purple) eQTL-SNPs by eGWAS. **c**: Per-chromosome percentage of genes whose transcription levels were linked to eQTL-SNPs. This percentage was calculated from the whole set of genes detected as expressed in blood. **d**: Percentages of genes associated with local-acting (red), distant-acting (blue) or both local- and distant-acting (purple) eQTL-SNPs. **e**: Per-chromosome percentage of ASE-SNPs of the whole set of 80,939 heterozygous SNPs in the group of 38 pigs included in the ASE analysis. **f**: Per-chromosome percentage of genes harboring at least one ASE-SNP in the set of genes with heterozygous SNPs in the group of 38 pigs

The analysis of eQTL genomic locations revealed that both local and distant associations were widely distributed on all chromosomes, although there were significant inter-chromosomal differences in the density of eGWAS signals (Fig. 2a to d). Chromosomes SSC1, SSC8, SSC11, SSC13, SSC15, and SSC16 had significantly lower proportions of eQTL-SNPs than the genome as a whole, while SSC4, SSC7, SSC12, and SSC14 were relatively enriched in eQTL-SNPs (one-sided Fisher <0.05). In addition, the relative proportions of local, distant, and local / distant eQTL-SNPs differed among chromosomes, especially on SSC1, SSC7, SSC12, and SSC15 (Chi^2^ < 0.05). We also observed that the relative percentage of associated genes varied among chromosomes (Fig. 2c). In particular, SSC1, SSC3, SSC6, and SSCX had lower percentages of associated genes than the genome as a whole (Fisher <0.05), while SSC7, SSC10, and SSC14 were found to be enriched in associated genes (Fisher <0.05). We also observed inter-chromosomal variation in the percentage of genes associated with local, distant, or local + distant eQTL-SNPs, as represented in Fig. 2d. In particular, SSC6, SSC12, SSC14, and SSCX presented a significantly different ratio between local- and distant-associated genes (Chi^2^ < 0.05) in comparison to the proportion in the whole genome (Additional file 3: Figure S1). From these results, we targeted a subset of the most promising regions and networks, according to their level of significance and connectivity or their relevance to immune response, for further analyses.

### ASE analysis confirmed extensive *cis*-regulation of gene expression in blood and revealed additional *cis*-regulatory events

ASE analysis was performed on whole-blood RNA-Seq data from a group of 38 Large White pigs. We found 12,811 Ensembl genes (release 82) expressed in blood; of these, 9501 were assigned a HUGO gene symbol. To be considered expressed, a gene had to be represented by a minimum of 10 reads in at least one sample. Since genome re-sequencing of the 38 pigs was not available, SNP identification was carried out using the RNA-Seq data. Thus, this analysis only detected SNPs with different expressed alleles in the samples included in the ASE study, and was not able to detect silencing events.

After SNP calling on RNA-Seq reads, 82,419 SNPs met the quality control criteria, including 71,365 (86.2%) SNPs already known in the dbSNP database. Among these SNPs, 80,939 were heterozygous in at least one of the 38 samples, and 86.3% of these were already known in dbSNP. Overall, 6824 genes were expressed and overlapped with at least one heterozygous SNP in one individual, and were thus detectable as affected by ASE.

From our analysis, 2286 SNPs showed allelic imbalance (Additional file 4: Table S3). These SNPs were further referred to as ASE-SNPs. Of these, 63.5% had already been described in dbSNP (1452 SNPs). They overlapped 1312 transcripts and 989 Ensembl (release 82) annotated genes, of which 763 had a gene symbol and could be further explored with functional enrichment analysis. Overall, these *cis-*regulated genes represented roughly 11% of all expressed genes that overlapped at least one heterozygous SNP. The genome localization of ASE-SNPs revealed that the candidate *cis*-regulated genes were widely distributed along chromosomes, with a great deal of inter-chromosomal variability. Specifically, enrichment in ASE-SNPs was detected on SSC5, SSC7, SSC9, SSC14, and SSCX, with the highest levels found on SSC7 (Fig. 2e). In addition, SSC1, SSC11, and SSC13 were significantly depleted in genes affected by ASE-SNPs, while SSC3 and SSC5 were significantly enriched (Fig. 2f, one-sided Fisher <0.05). The distribution of ASE-SNPs along each chromosome was not homogeneous (Additional file 5: Figure S2).

In total, 149 genes were detected by both the eGWAS and ASE analysis (Additional file 4: Table S3); this corresponded to 7% of the associated genes found by eGWAS, 20% of genes affected by ASE, and 2.7% of all genes detected by both approaches. Among these 149 genes, 109 were represented by a sense-probe and associated with a local eQTL-SNP by eGWAS. This result provided validation of the genetic *cis-*regulation of these genes. Fifty-two genes that were affected by ASE were also found by eGWAS to be associated with a distant eQTL-SNP, and of these, 16 had a distant association with an eQTL-SNP on another chromosome. These 16 genes were thus candidates for being both *cis-* and *trans*-regulators.

### Functional annotation of eQTL-SNPs, ASE-SNPs, and candidate regulated genes

The effects of eQTL-SNPs and ASE-SNPs were predicted using the Variant Effect Predictor (VEP) [21] and are summarized in Table 2. Of the eQTL-SNPs, 50.5% and 34% corresponded to intergenic and intronic variants, respectively. However, there were significantly fewer intergenic variants among eQTL-SNPs than in the SNP set as a whole (*i.e*. all SNPs that met QC criteria), while the opposite was true for intronic variants. With the ASE analysis, we observed that a majority of the detected SNPs with allelic imbalances corresponded to 3’UTR (24.5%), missense (23.6%), or synonymous (18.1%) variants.

**Table 2:** Summary of the effects of SNPs identified by eGWAS and ASE, and their relative proportions in the larger population of a given SNP category (%)

SNP effects	eGWAS			ASE
	QC-SNPs^a^	eQTL-SNPs	*P*-values^b^	ASE-SNPs
3’UTR variant	274 (0.65%)	47 (1.47%)	1.71 × 10^−6^*	560 (24.5%)
5’UTR variant	51 (0.12%)	5 (0.16%)	5.95 × 10^−1^	51 (2.2%)
Downstream gene variant	1278 (3%)	150 (4.7%)	9.03 × 10^−7^*	125 (5.5%)
Intergenic variant	27,211 (64.7%)	1606 (50.5%)	4 × 10^−56^*	150 (6.6%)
Intronic variant	10,893 (25.9%)	1083 (34%)	1.4 × 10^−22^*	209 (9.1%)
Missense variant	187 (0.44%)	28 (0.87%)	1.79 × 10^−3^*	539 (23.6%)
Non-coding exon variant	18 (0.04%)	3 (0.09%)	1.80 × 10^−1^	127 (5.6%)
Splice region variant	68 (0.16%)	7 (0.21%)	3.70 × 10^−1^	14 (0.6%)
Stop lost	7 (0.016%)	1 (0.03%)	4.42 × 10^−1^	5 (0.2%)
Stop retained variant	16 (0.038%)	4 (0.13%)	4.78 × 10^−2^*	1 (0.04%)
Synonymous variant	553 (1.31%)	68 (1.19%)	3.45 × 10^−4^*	414 (18.1%)
Upstream gene variant	1526 (3.63%)	182 (5.7%)	1.92 × 10^−8^*	51 (2.2%)
Mature miRNA variant	1 (0.002%)	0		1 (0.04%)
Splice acceptor variant	1 (0.002%)	0		10 (0.4%)
Stop gained	5 (0.012%)	0		17 (0.7%)
Splice donor variant				12 (0.5%)

^a^SNPs that met QC criteria

^b^ *P*-values for a Fisher test. For *P* < 0.05, the proportion of the SNP effect is significantly different between that of SNPs that passed the QC and that of eQTL-SNPs. Significant differences are labeled by a *

**Fig. 3:**
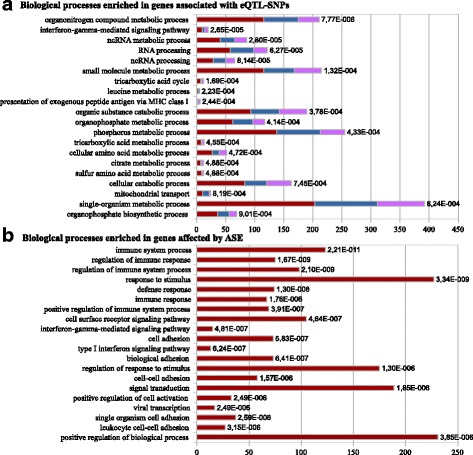
Biological processes enriched in genes associated with eQTL-SNPs (**a**) and/or affected by ASE (**b**). **a**: The top 20 biological processes enriched in genes associated with eQTL-SNPs by eGWAS. Horizontal bars represent the number of associated genes per biological process, with the corresponding q-values. Bars were split in order to represent the respective numbers of local-associated genes (dark red), distant-associated genes (blue), and local + distant-associated genes (purple). **b**: Biological processes enriched in genes affected by ASE. The horizontal bars represent the number of ASE-genes involved in each biological process, with the corresponding q-values. The two enrichment analyses were performed with the GOrilla tool

In order to explore whether the genetic control of gene expression in blood has an impact on specific biological pathways, we performed a functional enrichment analysis of the genes associated with local and/or distant eQTL-SNPs (Fig. 3a) and genes that contained an ASE-SNP (Fig. 3b). This analysis was carried out by first converting the pig gene lists into human gene lists using Biomart [22], in order to upload them into the GOrilla tool [23] to identify and visualize enriched gene ontology (GO) terms. For both eGWAS and ASE approaches, the top 20 most-enriched biological functions are presented in Fig. 3, with the full lists for eQTL in Additional files 6, 7 and 8: Tables S4, S5 and S6 and for ASE in Additional file 9: Table S7.

Among the top 20 most-significant biological processes that were enriched among genes associated by eGWAS with local and/or distant eQTL-SNPs (Fig. 3a), 12 were related to metabolic and catabolic processes and three to RNA processing (RNA and non-coding RNA). Other significantly enriched processes included the tri-carboxylic acid cycle, the cellular catabolic process, mitochondrial transport, and the organophosphate biosynthetic process. The two immunity-related functions on the list involved the "interferon-gamma-mediated signaling pathway" and the “presentation of exogenous peptide antigen via MHC class I, TAP-independent”, which was also among the top 20 enriched processes in the ASE analysis (Additional file 9: Table S7). In order to prevent bias in the enrichment analysis due to the inclusion of both local and distant associations, we also performed a separate enrichment study for each association type (Additional files 7 and 8: Tables S5 and S6, Fig. 3a). There were 18 GO terms enriched among genes that were associated with distant eQTL-SNPs, while 7 terms appeared on the list generated by an analysis of genes linked to local eQTL-SNPs. The only function present in both lists was the ncRNA metabolic process. No immunity-related functions were shared by both local and distant associations; instead, the “interferon-gamma-mediated signaling pathway” was enriched in local-associated genes and the “antigen processing and presentation of exogenous peptide antigen via MHC class I” function was identified only by combining all data. Three biological processes related to RNA processing were enriched among distant-associated genes. Overall, although a few processes were shared between genes associated with local or distant eQTL-SNPs, for the most part, the two groups of genes seemed to be involved in different biological pathways (Additional files 7 and 8: Tables S5 and S6 and Additional file 10: Figure S3). The high degree of overlap in enriched GO terms between the global analysis and the local-associated genes could be mostly due to the over-representation in the former analysis of genes associated with local eQTL-SNPs.

The genes that overlapped ASE-SNPs were significantly linked to a much higher number of biological functions than that identified from eGWAS data (Additional file 9: Table S7). In total, the ASE-genes corresponded to 105 enriched functions, with many of these related to immunity. Indeed, the top function listed among the 20 most-significant biological processes (Fig. 3b) was the immune system process (GO:0002376), followed by 12 other processes associated with immunity and defense, response to stimulus, or viral transcription, and 5 processes associated with adhesion and signaling pathways, which included both type I interferon and interferon-gamma-mediated signaling pathways. In addition, *cis*-regulated genes revealed by the ASE analysis were also found to be enriched for processes related to translational initiation (GO:0006413), nuclear-transcribed mRNA catabolic process nonsense-mediated decay (GO:0000184), and regulation of translation (GO:0006417), which could all be linked to the genetic control of gene expression, especially by master regulators. Two functions linked to heme metabolism and biosynthesis were also significantly enriched among the *cis-*regulated ASE genes (GO:0006783: “Heme biosynthetic process”, GO:0006783: “Heme metabolic process”), which is unsurprising for genes expressed in blood. Only two GO functions were shared between the list based on ASE genes and that produced using the eGWAS approach (Additional file 10: Figure S3): the "interferon-gamma-mediated signaling pathway" (GO:0060333) and “antigen processing and presentation of exogenous peptide antigen via MHC class I, TAP-independent” (GO:0002480).

### Construction of association networks revealed that genes are functionally linked and under shared genetic control

Since several eQTL-SNPs were associated with variation in the expression of more than one gene, and many genes were also linked to more than one eQTL-SNP (Table 1), we were able to construct potential regulatory networks by overlapping the eQTL signals. The whole set of SNP-probe associations was visualized in Cytoscape v3.2.1 [24], which identified 831 independent networks involving more than two nodes (Table 3). A network of three nodes represented either two eQTL-SNPs associated with the same gene or two genes associated with the same eQTL-SNP. Most networks were simple (655 consisted of one eQTL-SNP affecting several genes). When all networks were taken together, nodes had on average 1.5 neighbors, and we found 329 multi-edge node pairs, which represented associations between a single eQTL-SNP and multiple probes assigned to the same gene.

**Table 3:** Number and size of putative regulatory networks constructed from eGWAS-based associations

Number of nodes per network	3	4	5	6	7	8–154	Total
Number of networks	488	166	73	42	13	41	831

**Fig. 4:**
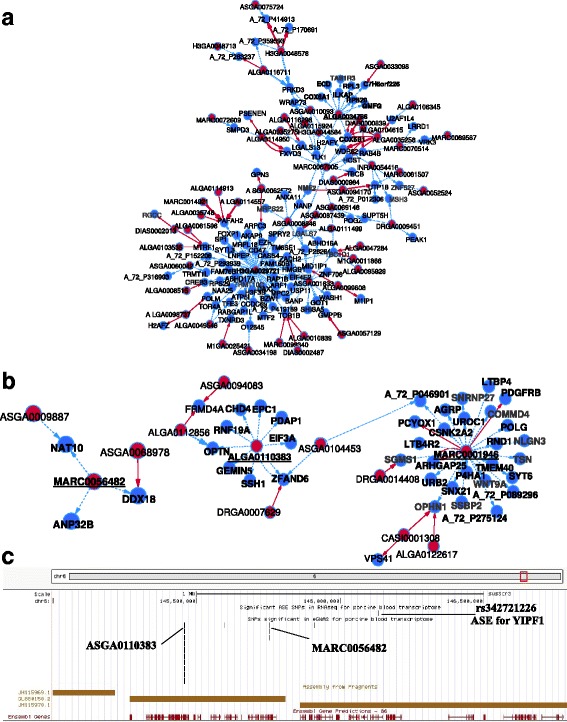
Visualization of association networks revealed by eGWAS (**a**, **b**) and links with ASE (**c**). Association networks were constructed based on multiple associations between eQTL-SNPs or genes, and were visualized by Cytoscapev3.2.1. eQTL-SNPs and genes are represented by red and blue dots, respectively. Associations are depicted with directed edges (oriented arrows) to show regulation by the eQTL-SNP genotype on gene transcription and not the reverse. Local associations are drawn with red arrows and distant associations with blue dotted arrows. All nodes correspond either to a gene or an eQTL-SNP, with the names indicated. Names in bold are further described in the results. **a**: The biggest association network drawn in this study comprises 154 nodes (eQTL-SNPs or genes). It is centered on two eQTL-SNPs: H3GA0029721 (rs81422644), which was distant-associated with 51 probes representing 47 genes, and ALGA0034786 (rs81393122). **b**: Visualization of the association networks centered on eQTL-SNPs MARC0001946, ALGA0110383, and MARC0056482. **c**: Map of a segment of SSC6 that comprises the ASE-SNP rs242721226 and the eQTL-SNPs ALGA0110383 and MARC0056482. The distances between the position of ASE-SNP rs342721226 and the eQTL-SNPs ALGA0110383 and MARC0056482 are 676 and 381 Kb, respectively

The largest network (Fig. 4a) was centered on eQTL-SNP rs81422644 (H3GA0029721), which mapped onto SSC10 at 30,039,892 bp (Additional file 1: Table S1). This eQTL-SNP was associated with the distant regulation of transcription in 51 probes, which were annotated to 47 distinct genes (three probes could not be annotated to any known porcine genes and for the *LNPEP* gene there were two probes). These 47 genes were widely distributed on all chromosomes, with the exception of SSC3 and SSCY (Additional file 11: Figure S4A). Using the R package PCIT [25], we applied the Partial Correlation and Information Theory algorithm [26] on expression correlations; this showed that variations in transcription levels of these genes were positively correlated regardless of SNP genotype (average correlation coefficient = 0.75; Additional file 11: Figure S4B). These analyses suggested that the 47 genes are genetically controlled by a common regulator in close vicinity to eQTL-SNP rs81422644. However, only two individuals in our analyses were homozygous for the alternative allele of this eQTL-SNP, which means that this potentially interesting master regulatory SNP must be confirmed using another pig population in which this allele is present at a higher minimum allele frequency (MAF).

**Fig. 5:**
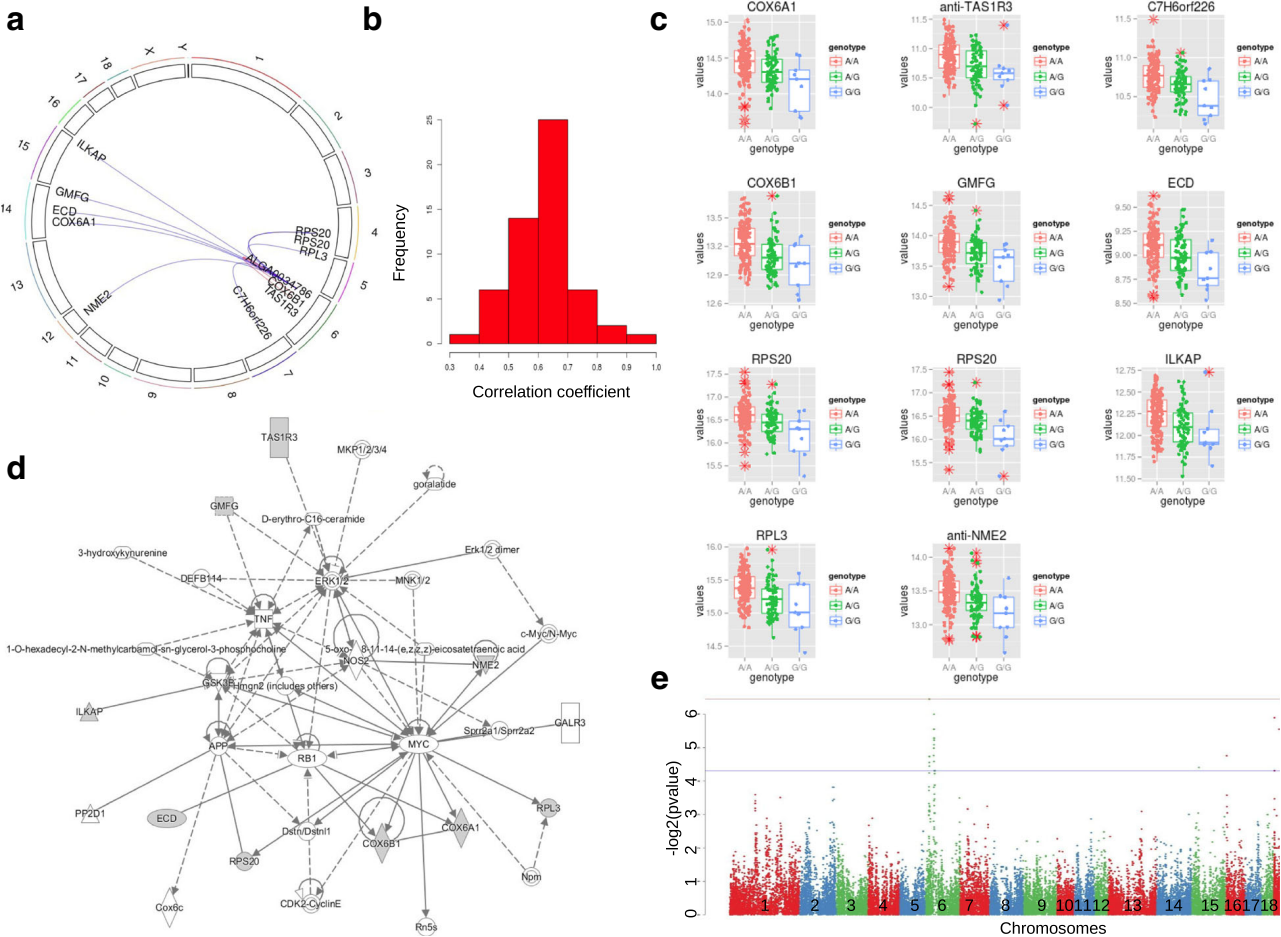
Detailed analysis of the association network centered on eQTL-SNP ALGA0034786 on SSC6. **a**: Circo plot produced by R package RCirco which maps eQTL-SNPs and associated genes according to their genomic positions. Links in red correspond to associations between the eQTL-SNP ALGA0034786 and genes mapping to SSC6, and links in blue represent associations with genes on other chromosomes. **b**: The distribution of all (grey) and significant (red) correlation coefficients calculated from pairwise comparisons of expression variation in probes associated with ALGA0034786, performed by PCIT. Red areas of the histogram correspond to coefficient correlations involving significant edges, as determined by PCIT, and gray areas represent non-significant edges. **c**: Boxplots of expression variations in probes annotated for porcine genes, depending on the eQTL-SNP genotype. Each dot represents one animal. Pink dots correspond to pigs homozygous for the most-frequent allele (genotype A/A), green dots to heterozygous pigs (genotype A/G), and blue dots to animals homozygous for the less-frequent allele (genotype G/G). **d**: Ingenuity Pathway Analysis of the genes included in the association network. The functional network comprised nine of the ten genes that were distant-associated to eQTL-SNP ALGA0034786. The symbols of these nine genes are colored in gray. The IPA-drawn network revealed functions linked to “cellular assembly and organization”, “cell death and survival”, and “endocrine system disorder”. **e**: eGWAS results for probe A_72_P211467, which was annotated for the gene *COX6B1*, visualized with a Manhattan plot. The association peak on SSC6 corresponds to the eQTL represented by eQTL-SNP ALGA0034786. The red horizontal line corresponds to an FDR threshold <0.01 and the horizontal blue line to an FDR threshold <0.05

This large network was connected to another network that was centered around eQTL-SNP rs81393122 (ALGA0034786), which was located on SSC6 at 19,761,160 bp (Additional file 1: Table S1). This eQTL-SNP was linked to the distant regulation of 11 probes, annotated for ten genes on six distinct chromosomes (Fig. 5a, Additional file 1: Table S1): ribosomal protein L3 **(** *RPL3*), ribosomal protein S20 (*RPS20*), cytochrome c oxidase subunit Vib polypeptide 1 (*COX6B1*), cytochrome c oxidase subunit VIa polypeptide 1 (*COX6A1*), non-metastatic cells 2 protein (*NME2*), taste receptor, type 1, member 3 (*TAS1R3*), integrin-linked kinase-associated serine/threonine phosphatase (*ILKAP*), glia maturation factor gamma (*GMFG*), uncharacterized protein C7H6orf226, and ecdysoneless cell cycle regulator (*ECD*). PCIT analysis revealed positive correlations among transcription variations in the 11 probes, with an average correlation coefficient of 0.61 (Fig. 5b). We also observed that this eQTL-SNP genotype affected the transcription of the ten genes in the same direction (Fig. 5c). Strikingly, nine of the ten associated genes were included in a unique IPA-predicted network related to the functions “cellular assembly and organization”, “cell death and survival”, “endocrine system disorders” (Fig. 5d). One of the most significant associations in the network was with the gene *COX6B1* (Fig. 5e).

As represented in Fig. 4b and c, a candidate master regulatory SNP (rs81288717, or MARC0001946) was detected in an intergenic hotspot on SSC3, at 76,408,626 bp (Additional file 1: Table S1). This eQTL-SNP was associated with the distant-regulation of transcription variation in 26 genes, distributed on 14 different chromosomes (Additional file 12: Figure S5A). The eQTL-SNP genotype affected the transcription of the 26 genes in different directions, but with additive effects. As an example, allele G was associated with increased transcription of the gene *UROC1*, but with decreased transcription of the gene *ARHGAP2* (Additional file 12: Figure S5B). Complementarily, PCIT analysis revealed that transcription variations in these 26 genes were strongly correlated, either positively (average correlation coefficient = 0.74) or negatively (average correlation coefficient = 0.47) (Additional file 12: Figure S5C). IPA highlighted that the 26 genes could be included in three different networks associated with immunity-related processes: ten genes were linked to “cellular development, hematological system development and function, hematopoiesis”, nine genes to “inflammatory response, cell signaling, molecular transport”, and five genes to “developmental disorder, hereditary disorder, organismal injury and abnormalities” (Additional file 12: Figure S5D). Thus, eQTL-SNP rs81288717 could be an interesting candidate genetic marker for the regulation of immunity-related genes in blood, but further work is needed to identify the causal genetic variant.

For a few networks, the central eQTL-SNP was located within 1 Mb of an ASE-SNP. As an example, we present the case of two eGWAS signals that were 295 Kb apart, both within 500 Kb of the same ASE-SNP (Fig. 4c). The eQTL-SNP rs81338631 (ALGA0110383) maps onto SSC6 at 145,457,494 bp, inside an intron of *PCSK9*, while eQTL-SNP rs81244817 (MARC0056482) is located on the same chromosome at 145,752,528 bp (Additional file 1: Table S1). The nearby ASE-SNP, rs342721226, is contained within the 3’UTR region of the *YIPF1* gene, at 146,133,708 bp (Fig. 4c). Association networks revealed that eQTL-SNP rs81338631 was linked to the distant-regulation of nine different genes located on seven distinct chromosomes, while eQTL-SNP rs81244817 was associated with three genes located on three different chromosomes (Additional file 13: Figure S6A). In a PCIT analysis, the transcription levels of genes associated with these two eQTL-SNPs were strongly positively correlated (0.63 on average; Additional file 13: Figure S6B). Moreover, IPA revealed that the whole set of genes associated with the two eQTL-SNPs, as well as the ASE-gene *YIPF1* and *PCSK9*, the gene that contained the eQTL-SNP rs81338631, could be included in a unique functional network related to “cell death and survival”, “organismal injury and abnormalities” and “tissue morphology” (Additional file: Figure S6C). Both eQTL-SNPs affected the transcription of their associated genes in the same direction (Additional file 13: Figure S6D). Overall, these two regulatory networks seem to be related and the ASE affecting *YIPF1* designed this gene as an interesting candidate for understanding the molecular mechanism of transcriptome regulation of associated genes (*YIPF1* is associated to GO annotations that include RNA binding and ribonuclease activity).

### A high density of local- and distant-acting eQTLs in the swine Leucocyte antigen (SLA) complex on SSC7

Chromosome SSC7 was enriched in both eQTL- and ASE-SNPs (Figure 2, Additional files 3 and 5: Figures S1 and S2). In particular, we observed numerous local and distant associations involving genes and SNPs within the MHC region, as has already been reported for human MHC [27–29].

**Fig. 6:**
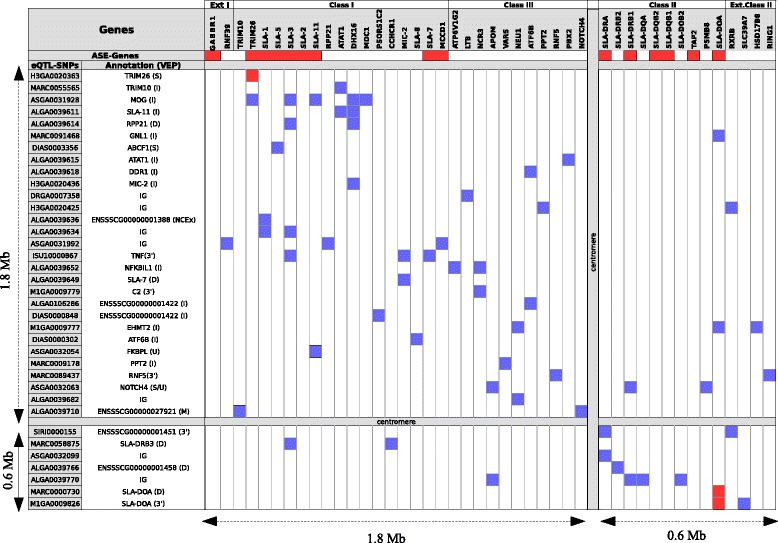
Representation of associations and ASE-SNPs mapping to genes within the SLA region. SNPs detected as eQTL-SNPs by eGWAS are listed on the y-axis and their VEP annotation is indicated. These SNPs are ordered according to their physical position on SSC7, using the SusScrofa.10.2 genome assembly as a reference sequence. The genes listed along the x-axis are those that were found to harbor ASE-SNPs already referenced in dbSNP or that were associated with eQTL-SNPs by eGWAS. These genes are ordered using the map of the Hp1a.1 haplotype as a reference sequence [30]. All genes affected by ASE are indicated with red boxes and all distant associations are indicated with blue boxes. The centromere known to map between the class III and II sub-regions is shown. I = Intronic variant; IG = Intergenic variant; D = Downstream variant; U = Upstream variant; 3′ = 3’UTR variant; S = Synonymous variant; NCEx = Non-coding exonic variant; M = Missense variant

The MHC in pigs is referred to as the SLA complex and maps to SSC7 on both sides of the centromere; the class I and III subregions cover 1.8 Mb of 7p1.1 band while the class II subregion spans 0.6 Mb of 7q1.1 band, with the size of the in-between centromere as-yet unknown [30]. Within this relatively small region, the SLA complex contains 151 annotated gene loci, and due to this high gene density, our previous definition of local/distant associations (inside/outside a 2-Mb window centered on the expressed probe) was not appropriate. Indeed, for the majority of eQTLs that mapped to the SLA complex, we could not distinguish between local and distant associations. Figure 6 illustrates the associations identified between gene expression levels and eQTL-SNPs, as well as genes affected by ASE-SNPs that have already been described in dbSNP. According to our eGWAS results, the expression levels of 51 genes within the SLA complex were found to be genetically controlled by 78 eQTL-SNPs, which included 11 SNPs located on chromosomes others than SSC7 and 31 SNPs on SSC7 but outside the SLA region. We identified 36 non-redundant eQTL-SNPs within the SLA complex and their VEP annotation is summarized in Fig. 6. Of these 36 eQTL-SNPs, 29 mapped to annotated genes, which included four SLA genes (*SLA-7*, *SLA-11*, *SLA-DRB3,* and *SLA-DOA*) and 23 non-SLA genes. The remaining two were considered to be intergenic when only the most severe consequence of each SNP was considered in VEP analysis.

Twelve SLA genes and 39 non-SLA genes were found to be regulated by between one and five distinct eQTL-SNPs. Among those affected were four functional SLA class I genes, the classical class Ia genes *SLA-1* and *SLA-3*, as well as two non-classical class Ib genes, *SLA-7* and *SLA-8*. In the class II subregion, the expression levels of most genes were found to be genetically regulated, including *SLA-DRA*, *SLA-DRB1*, *SLA-DRB2*, *SLA-DQA*, *SLA-DOB2*, and *SLA-DOA*, as well as the non-SLA genes *TAP2* and *PSMB8*. All these data revealed that transcriptional activity is both dense and locally regulated. eQTL-SNPs were found to act either within a sub-region or more distantly, on genes mapping to both sides of the centromere. Only *SLA-DOA* and *TRIM26* were associated with an eQTL-SNP that had been annotated as a variant for these genes: a 3’UTR variant for *SLA-DOA* and a synonymous variant for *TRIM26*. These two genes were thus identified as good candidates for *cis-*regulation. We did not find eQTL-SNPs outside of SSC7 that were associated with changes in the expression of SLA genes; the only associated eQTL-SNPs were located either within the SLA complex or in close vicinity. We detected 32 eQTL-SNPs specifically associated with a single gene (*SLA-1*, *SLA-3*, *SLA-5*, *SLA-8*, *SLA-11*, *SLA-DRA*, *SLA-DRB2*, or *SLA-DOA*). These SNPs could thus be considered genetic markers that specifically target the expression of one of these genes.

Through ASE analysis, we identified nine genes within the class I subregion and six genes within the class II subregion that harbored putative ASE-SNPs (Fig. 6). Interestingly, each SLA gene was found to be affected by specific ASE-SNPs. *SLA-DQB1* and *SLA-DQB2* shared the same three ASE-SNPs because of their close vinicity. The number of ASE-SNPs per SLA gene varied. *SLA-3* harbored 84 SNPs with allelic imbalance, while *SLA-2* and *SLA-DRB1* showed 27 and 28, respectively. *SLA-DOA* and *SLA-DRA* harbored only one ASE-SNP. This observed variation in the number of SNP variants per SLA gene is consistent with the expected allelic variability according to each gene (www.ebi.ac.uk/ipd/mhc/group/SLA). The functional classification of ASE-SNPs for SLA genes is presented in Table 4.

**Table 4:** Number and annotation of ASE-SNPs affecting MHC genes

	SLA-1	SLA-2	SLA-3	SLA-5	SLA-7	SLA-11	SLA-DRA	SLA-DRB1	SLA-DOA	SLA-DQB1/2
3’UTR variant	13	15				6	1			3
5’UTR variant						1				
Intron variant	17	5	13		2			2		
Misssense variant	28	2			1	7		14		
Non-coding transcript exon variant	2		61	12						
Splice region variant	12	1	1							
Synonymous variant						3		6	1	
Downstream gene variant		4	12		3	3		6		
Intergenic variant										
Total^a^	72	27	84	12	3	20	1	28	1	3

^a^ Total of ASE-SNPs per gene identified in the 38 pigs included in the analysis. The total numbers do not correspond to the sum of annotated SNPs because a few SNPs were annotated differently depending on the transcript they overlapped

### Overlap of eQTL-SNPs and ASE-SNPs with blood biomarkers previously identified in pigs

We compared the lists of genes found using the ASE or eGWAS datasets to lists of genes that we previously reported to be differentially expressed (DE) in the blood of pigs; this included results from in vitro production of IL-2 and IL-10 after stimulation of total blood, in vitro phagocytosis tests, and counts of T lymphocytes CD4-CD8+ [4]. For each immunity-related trait, numerous DE genes were associated with eQTLs or ASE (Table 5), indicating that expression levels of blood biomarkers for these traits are genetically controlled. For example, local eQTL-SNPs were associated with expression changes in the *ALOX12* gene, which has been identified as a blood biomarker for phagocytosis, as well as the genes *GZMB* or *KLRG1*, which are correlated CD4-CD8+ cell count. Instead, two distant eQTL-SNPs were implicated in the transcription variation of *GATM*, which has been identified as a candidate biomarker for phagocytosis level; these eQTL-SNPs mapped less than 3 Mb from *GATM*. Finally, for CD4-CD8+ cell count, the candidate blood biomarkers *TNL1*, *GNLY*, *GZMB*, and *KLRG1* were found to be affected by a SNP with allelic imbalance.

**Table 5:** Correspondence between sets of eQTL- and ASE-SNPs detected in this study and gene sets that were differentially expressed (DE) in the blood of pigs studied for levels of immunity traits

	IL-2^a^	IL-10^b^	Phagocytosis^c^	CD4-CD8 + ^d^
DE genes [3]	850	733	1195	52
DE local-associated genes	69	64	107	5
DE distant-associated genes	56	47	74	0
DE genes with ASE signal	48	29	61	7

^a^Production of IL-2 after in vitro stimulation of total blood with phorbolmyristate acetate (PMA) and ionomycin for 48 h

^b^Production of IL-10 after in vitro stimulation of total blood with PMA and ionomycin for 48 h

^c^Quantification of phagocytosis capacity via in vitro tests on total blood

^d^CD4-CD8+ lymphocyte count measured by flow cytometry

## Discussion

### eGWAS and ASE analysis provide complementary results for mapping genetic regulation of gene transcription in blood

In the current study we investigated the genetic control of the blood transcriptome in 60-day-old pigs by combining two high-throughput methods: i) an eGWAS which analyzed transcription in 242 pigs (130 uncastrated males and 112 females) using expression microarrays and genome-wide SNP genotyping, and ii) an evaluation of ASE in 38 castrated males based on RNA-Seq of total blood RNA. The groups of animals used for the eGWAS and ASE analysis were independent from each other but selected from the same larger experimental population of Large White pigs, and partly generated from common boars. In doing this, we aimed to prevent discrepancies in the results due to heterogeneity in genetic backgrounds. Each approach had a different focus: eGWAS maps eQTLs as genomic loci that influence levels of gene transcripts through local and/or distant actions [8], while ASE analysis identifies allelic imbalance among transcripts. In our study, eQTL-SNPs were defined as acting locally or distantly based on whether the SNP was less or more than 1 Mb from either side of the start position of the associated probe, respectively. Globally, we found 2124 local eQTL-SNPs which affected the expression of 1363 genes, and 1187 distant eQTL-SNPs affecting 1010 genes. In parallel, ASE analysis enabled us to detect 2286 candidate SNPs that showed allelic imbalance, and which affected 763 genes.

Both eGWAS and ASE analyses were performed using stringent parameters to ensure robust mapping. For eGWAS, we chose to simultaneously analyze local and distant eQTLs [31–33], while previous studies had performed two independent association analyses which separated local- and distant-acting SNPs [27, 29, 34]. Due to the large amount of transcript information and the number of SNPs involved, eGWASs require extremely extensive calculations, which can raise issues related to statistical power [32]. To address this, we used stringent quality control thresholds, and corrected local and distant associations for multiple testing with an FDR procedure at two levels: i) for all association tests for each probe, and ii) for all association tests for all probes. For ASE analyses we filtered out SNPs with fewer than 10 reads and retained only ASE-SNPs with significant allelic imbalance in their read abundances in at least one-third of heterozygous animals.

For both eGWAS and ASE approaches, we observed that the genetic control of gene transcription was heterogeneously distributed along the genome, with important variations among chromosomes in both numbers of eQTL- or ASE-SNPs as well as in the relative proportions of genes that demonstrated ASE or that were associated with eQTLs. However, we found only limited overlap between the local associations that were identified by eGWAS and the *cis-*associations that were identified by the ASE analyses, which is consistent with results from other studies [10, 11]. Indeed, this was expected due to inherent differences between the approaches and their respective limits. For eGWAS, only eQTLs that corresponded to SNPs represented on the 60K SNP chip could be detected and in this design, intergenic SNPs are overrepresented [35]. Consequently, the functional annotation of eQTL-SNPs remains limited, and they mainly reflect cases of linkage disequilibrium with nearby causal variants and not direct causal links. The ASE analysis was limited by the lack of whole-genome sequence data for the animals included in this analysis, which meant that SNPs were called using RNA-Seq data. Therefore, in order to limit errors in SNP discovery, only ASE-SNP positions for which both alleles were expressed (Minor Allele Count >3) were considered in the final analysis, and transcripts with low read counts were discarded. In addition, due to the lack of parental information, we could not differentiate between allelic imbalance and parental imprinting. However, among the 763 ASE genes found in our study, only three are described as imprinted genes in the Geneimprint database (http://www.geneimprint.com/): *SNRPN*, which is imprinted by the paternal allele in *Homo sapiens*, *Macaca mulatta*, and *Bos taurus*; and *SNORD64* and *DMTN*, which are reported as imprinted only in *Homo sapiens*.

Overall, all these limitations suggest that these eQTL-SNPs and ASE-SNPs are not causal variants but SNPs in linkage disequilibrium with the causal variants that could be used as genetic markers for gene expression in the pig population included in our study. We did not address the question of sex effect on blood transcriptome variation but we included the sex as a cofactor in the eGWAS models and used only castrated males for the ASE analyses.

In a comparison of ASE and eGWAS results, 149 associated genes in eGWAS overlapped with ASE-SNPs. For genes found *cis*-associated in eGWAS, ASE analysis validated the putative *cis-*regulation of their expression. By combining eGWAS and ASE analyses, we were able to more extensively map the genetic control of the blood transcriptome and this approach will likely contribute to a more fine-scale mapping of the candidate causal polymorphisms. The heterogeneity of associated SNPs along the genome can be observed with greater precision using ASE approaches than with eGWAS, and this precision enabled us to pinpoint the most relevant genomic regions, as illustrated by the example of the SLA region. At the functional level, we found that ASE-genes were more enriched in biological functions specifically related to immunity than the genes locally or distantly associated with eQTL-SNPs were. However, the few enriched GO terms that were shared between the two analyses were immune-related. The GO results were complemented by the fact that the ASE analysis pinpointed immune functions as one of particular phenotypes affected by gene expression found under genetic control. These findings suggest that ASE results are more precise and accurate than eQTL results. In our study, this difference could be related to the relatively low density of SNPs in the chip used for eQTL mapping. Finally, ASE can provide information relevant in refining the mechanism of genetic regulation of gene expression by completing for instance putative regulatory networks.

### Results are consistent with previous eQTL studies of human whole blood

Globally, our results provided general patterns of eQTL mapping in blood that were consistent with previous results on whole blood in humans. First, we detected 1.6 times more local associations (2839) than distant associations (1751), an imbalance that has already been reported in previous studies [12, 31, 33, 36]. This could be because distantly acting regulators have weaker effects than locally acting ones, resulting in associations that require greater statistical power for detection. Moreover, *trans*-eQTLs are more cell specific than *cis*-eQTLs [29] and because the heterogeneous nature of blood, *trans*-signals will be weaker. Although we cannot preclude the explanation that the larger number of local associations recovered here is due to a lack of statistical power to detect distant/*trans*-regulation, our results suggest that the number of local/*cis*-regulators truly does exceed the number of distant/*trans*-regulatory SNPs.

We observed that local eQTLs, distant eQTLs, and *cis*-regulators were widely distributed across all chromosomes, as has been previously described [31, 33]. Interestingly, nine genes that were associated with local eQTL-SNPs, two of which belonged to the *TMEM* family, had also been listed among the top 25 *cis*-associated genes reported by Joehanes et al. [12]. Likewise, a set of 324 *cis-*acting genes reported by Mehta et al. [33] in a study on whole blood also contained 32 of the *cis*-associated genes found here. Moreover, as has also been reported in humans, we were able to identify putative candidate master regulatory variants, despite the heterogeneous nature of peripheral blood [28, 29, 31]. We detected at least 13 master regulatory loci, as represented by eQTL-SNPs that were associated with between 10 and 51 probes. These eQTL-SNPs influenced the expression of various genes, mostly in the same direction, as illustrated here by the examples of two SNPs, ASGA0110383 and MARC0056482. However, most master regulators were not associated with local genes, which was consistent with the results of a previous study [31].

We paid special attention to the SLA region on chromosome SSC7 because it harbored the greatest concentration of genetic regulatory elements in both eGWAS and ASE analyses. Previous studies in humans have reported similar findings. For example, Fairfax et al. [29] found that several loci in the MHC showed significant *trans*-associations. Examination of the MHC also revealed numerous eQTL-SNPs linked to complex diseases [27]. In addition, an eGWAS performed on blood from cohorts of healthy women and breast cancer survivors showed an association between ten HLA genes (*HLA-C*, *HLA-E*, *HLA-F*, *HLA-G*, *HLA-H*, *HLA-DPB1*, *HLA-DQA1*, *HLA-DQB1*, *HLA-DRB3*, *HLA-DRB4*) and SNPs in 100 genes located on human chromosome 6 [37]. In particular, *CIITA* has been identified as a master *trans*-activator, with an essential role in initiating transcription of MHC class II genes in human stimulated B cells and monocytes [38]. However, our eGWAS did not generate any evidence of *CIITA* acting as a master regulator, despite the presence of one *CIITA*-related SNP on the array. In the ASE analysis, *CIITA* was expressed in the RNA-Seq data, but had no significant allelic imbalance. A possible explanation for this discrepancy is that such associations could be specific to conditions of immune stimulation and are not significant in a basal health state. Strikingly, we observed that almost all functional SLA class I and II genes harbored allelic imbalance, suggesting that fine-scale modulations of the expression levels of histocompatibility class I and II molecules may depend on the relative abundance of alleles. More in-depth analyses are required to determine whether this allelic imbalance affects peptide presentation efficiency and is associated with different immunity phenotypes.

Our results seem to be globally consistent with studies on the whole blood transcriptome in humans [28, 29, 31]. However, additional studies are needed on whole blood in pigs to assess the robustness of the list of local eQTL-SNPs reported here and to start to establish comparative eQTL maps where relevant. Thus, our work is a first step in generating useful resources for the study of eQTLs in pigs, as has been established in humans [12].

### A step forward in the characterization of blood biomarkers, particularly for immune capacity

Peripheral blood contains various types of important immune cells. Schramm et al. [31] demonstrated reasonable concordance between the *cis*-eQTLs found in their own study of whole blood and those reported in prior studies of primary monocytes or blood-derived lymphoblastoid cell lines. They thus confirmed that whole-blood eQTL studies are a good resource for the discovery of biomarkers, especially in the context of disease in humans. With the goal of improving selection for health traits in pigs, blood is also a relevant surrogate tissue for phenotyping immune capacity [39]. As shown in human studies, whole blood can be used for robust eQTL analysis despite its heterogeneous cell composition. Indeed, Mehta et al. [33] demonstrated that variations in cell count would exert only a minor effect on expression levels, as eQTLs are consistently replicated between whole blood and independent human cohorts.

We showed a notable degree of overlap between the genes found in this study to be genetically controlled and the QTLs available in the AnimalQTL database [40] for pigs. More precisely, we found overlap between the lists of eQTL- and ASE-SNPs generated here and QTLs of the AnimalQTL database that had been associated with immune-related phenotypes, including interferon-gamma level, IgG level, lymphocyte count and other traits related to blood function (Additional files 14 and 15: Table S8 and Table S9). In addition, we identified the genetic regulation of several previously published blood biomarkers for immunity traits (e.g., *ALOX12* for phagocytosis, *GZMB* and *KLRG1* for CD4-CD8+ cell count) [4], which suggests that these biomarkers could be used as molecular expression phenotypes in genetic selection efforts.

The integration of eGWAS- and ASE-based approaches, together with GWAS data when relevant, facilitates the identification of candidate genes and polymorphisms that are related to complex traits. Similar integrated approaches in pigs have been recently published for complex traits related to meat properties [14, 16], as well as for blood lipid traits linked with cardiovascular diseases [41] and traits associated with coping behavior [20]. In addition, this approach could help to pinpoint the biological and molecular bases of phenotype-genotype links that are highlighted by a GWAS-based approach. Integrated methods are also expected to be more accurate in the detection of loci under genetic control as the link between genotype and transcriptome is more direct than the link between genotype and end phenotype. These techniques can thus help to prioritize the study of candidate variants linked to changes in the transcriptome and phenotype.

## Conclusions

This study is, to the best of our knowledge, the first genome-wide analysis of the genetic control of gene expression in blood in the pig, based on a cohort of 60-day-old French Large White pigs. The combination of eGWAS and ASE results showed extensive genetic regulation of gene transcription, and provides a relevant resource for the study of genomic regions and variants linked to gene expression variation in pig blood. All results are available in a public Track Hub entry that can be explored using the genome browsers of Ensembl and UCSC. Additional studies are needed to cover more ages, breeds and environments in order to build an even more extensive map of genetic regulations of gene expression in blood. Overall, these data and approaches should contribute to future efforts to decipher the ultimate molecular mechanisms behind the genetic determination of pig phenotypes, especially for immunity- and health-related traits.

## Methods

### Animals and blood sampling

Two groups of pigs from the same French Large White selected line were bred either on a test farm at Le Rheu (38 pigs, IMMOPIG project) or on an INRA experimental farm at le Magneraud (243 pigs, SUS_FLORA project). The animals were partly generated from common boars and were weaned at 28 days of age. The group of 38 pigs belongs to a population of castrated males that was described in Flori et al. (2011) [2]. The group of 243 pigs comprised 131 uncastrated males and 112 females from a larger cohort that is described in Ramayo-Caldas et al. (2016) [42]. Details on the pig feed diet are available in Mach et al. (2015) [43] and correspond to standard nutritional practices in pig production. Peripheral blood (jugular vein) was sampled from 60-day-old pigs using EDTA-coated tubes and PAXgene Blood RNA tubes (PreAnalystiX, Qiagen, Germany). Blood samples were stored at −20 °C (EDTA tubes) or −80 °C (PAXGene tubes) prior to DNA or RNA extraction procedures, respectively. Complete blood cell counts were recorded for a subset of 195 pigs from the group of 243 pigs (Additional file 16: Table S10), in agreement with our observations that the animals showed no clinical signs of infection. Information on production traits are available in Sanchez et al., (2014) [44] for the ASE pig group and in Ramayo-Caldas et al. (2016) [42] for the eQTL pig group.

### Total RNA extraction from blood

Total RNA from blood was isolated using the PAXgene Blood RNA Kit (Qiagen, Germany) following the manufacturer’s instructions. RNA purity and concentration were determined using a NanoDrop 1000 spectrophotometer (Thermo Scientific, USA), and RNA integrity was assessed using the Bioanalyzer 2100 (Agilent Technologies, USA). RNA samples used for RNA-Seq had RINs ranging from 6.7 to 8.6 with 28S:18S ratios ranging from 0.8 to 1.6. RNA samples used for microarrays had RINs between 6.1 and 9.7.

### SNP genotyping

DNA was extracted from blood samples and genotyped using the Illumina PorcineSNP60 DNA chip which contains over 60,000 SNPs [35]. Genomic DNA extraction and SNP genotyping were carried out with the Labogena-DNA platform (Jouy-en-Josas, France). The initial quality control (QC) step of genotyping was performed using the R package GenABEL [45] and included heterozygosity testing and multidimensional scaling to identify population outliers. For SNP filtering, we used sample and SNP call rates >95%, a MAF > 5%, a Hardy-Weinberg equilibrium threshold corrected by FDR of 1%, and a XXY call of 0.8. We removed one individual that did not meet these QC criteria, resulting in 242 individuals (130 males and 112 females) with data available for 44,281 SNPs.

### Analysis of blood transcriptome using expression microarrays

Total blood RNA (200 ng) was retro-transcribed, Cy3-labeled, and hybridized onto customized single-channel 8X60K Agilent Technologies arrays (platform Agilent-037880/INRA *Susscrofa*60K v1), which were enriched in genes linked to immunity and muscle physiology, following the manufacturer’s instructions and a protocol described in Jacquier et al. (2015) [46]. Because genomic sequencing data were not available, it was not possible to take a possible effect of within cDNA target polymorphisms on hybridization levels into account. Pre-processing and quality control were performed by the Limma R package [47] on 60,306 probes. In brief, we first normalized the background by the normexp method. Then, intensities quantified by median signals were normalized by the quantile method. Probes were filtered according to their intensities in order to eliminate unexpressed genes; to be retained, probes had to be expressed at levels at least 10% higher than those of the negative controls on at least four arrays. In total, 59,774 probes that passed the quality control and the filtering step were considered to be expressed in the blood samples included in this study.

### Refinement of probe sequence annotation of the Agilent-037880/INRA *Susscrofa*60K v1 platform

Probe mapping and annotation were updated to refine all genomic locations. The 60-mer sequence probes included in the Agilent-037880/INRA *Susscrofa*60K v1 platform were mapped onto the pig reference genome (SusScrofa v10.2) with the TopHat aligner (v2.0.14) [48]. Allowing two mismatches and two gaps per probe sequence, we found 44,326 probes mapping to unique genomic positions. Among these, 41,839 probes were expressed in our samples. The annotation of these probes was performed by combining annotation information provided by the Ensembl (release 79) and NCBI (GCF_000003025.5) databases.

### Expression genome-wide association study (eGWAS)

The eGWAS was carried out on data from 242 pigs and linked SNP genotypes, based on animal genotypes obtained with PorcineSNP60 DNA chips, to blood gene-transcript levels, as detected by transcriptome analysis using Agilent-037880/INRA *Susscrofa*60K v1 microarrays. The association studies were performed with the GenABEL package [45] using the family-based score test for association [49], a polygenic mixed model to estimate the SNP effect, and a *p*-value in two steps. First, the polygenic additive model and likelihood were estimated using available data, and then the FASTA test statistic was computed using the maximum-likelihood estimates of the intercept, the proportion of variance explained by the polygenic component, and the residual variance. The model was corrected for different co-factors, with batch and sex as fixed effects, and with the familial structure of the sample as a random effect, represented by the genomic kinship matrix estimated using autosomal data. The individual variability in blood cell counts was not included in the analysis model since the data were not available for all the animals. Nonetheless, we observed a limited variability of these blood parameters within the population (see Additional file 16: Table S10). All probes and SNPs were treated independently for the first step of the association study. Next, multiple testing corrections were conducted at two levels. First, for each probe, *p*-values of the associations between the expression variation of that probe and all SNPs were corrected by an FDR procedure that controlled the overall type-I error rate at 5%. Secondly, to assess the significance of associations of all probes, a second significance threshold was calculated by FDR (5%) for the *p*-values of all associations. Only associations with a FASTA *p*-value lower than these two thresholds were considered significant.

Finally, in order to facilitate the global interpretation of results, we implemented additional association tests to limit redundancy. We reduced the definition of an eQTL to a representative SNP, i.e. for each eQTL region, we kept only the most significant associated SNP to represent the eQTL, referred to hereafter as an eQTL-SNP. In order to eliminate other significant associated SNPs in a 5 Mb window around a designated eQTL-SNP without missing an independent eQTL, we repeated the association analysis between the probe and these SNPs, correcting the polygenic mixed model by the effect of the most significant SNP, as has been reported in previous studies of human data [29, 50]. If the *p*-values associated with a given SNP did not reach the previously calculated significance threshold, the SNP was considered to have the same effect as the most significant SNP (the previously designated eQTL-SNP); it was thus considered redundant and removed from further analyses.

### Allele-specific expression (ASE) analysis by RNA-Seq

Sequencing libraries from total blood RNA (1.5 μg) were prepared using the TruSeq RNA Sample Preparation Kit (Illumina, San Diego, USA) according to the manufacturer’s instructions at INRA’s GetPlage platform (Toulouse, France) and as previously reported in Mach et al. (2014) [51]. Tagged cDNA libraries were sequenced on 2.66 lanes of a HiSeq2000 (Illumina) in 100-bp single-end reads. Then, raw reads were aligned to the pig reference genome (v10.2) with the STAR 2.4.0 aligner using an approach that comprised two alignment steps, as recommended by the Broad Institute (https://software.broadinstitute.org/gatk/guide/article?id=3891). First, splice junctions were detected by a first pass of STAR mapping. Detected splice junctions were then used during a second round of mapping to take into account the discontinuous nature of the RNA sequencing data, allowing only one mapping position per read. STAR includes a soft-clipping step, which takes care of adapter contamination. After the two-pass procedure, we used a GATK (v3.4, [52]) tool called SplitNCigarReads, which split reads into exon segments and hard-clips any sequences overhanging into the intronic regions to reduce the number of called false variants. Then, reads were realigned around indels to limit artifacts due to the alignment algorithm. Finally, the standard GATK base recalibration procedure re-adjusted the quality scores assigned to the individual base calls in each sequence read, thus reducing the systematic technical error linked to the sequencing machines.

In order to prevent false positive ASE signals due to allelic mapping biases that could influence allele counts, variants were called in the dataset, filtered using vcftools (v.0.1.12a), and then nucleotides that were characterized as SNPs were masked in the reference genome with maskFastaFromBed (Bedtools v2–2.24.0). Reads were then realigned on the masked reference genome using the same procedure as described previously in order to have the same constraint of one mismatch for reads that had either of the two alleles at the SNP position.

Allele counts were performed using a GATK (v.3.4) tool called ASEReadCounter which calculates allele counts at SNP positions. A binomial test determined whether there was a significant difference in abundance of each allele of a SNP. We corrected *p*-values on a per-animal basis using the FDR procedure (5x10^−2^). Only bi-allelic SNPs with a significant differential abundance in alleles in more than 1/3 of heterozygous animals for the considered SNP were analyzed further.

### Analysis of function enrichment and SNP annotation

A functional enrichment analysis of genes associated with eQTL- or ASE-SNPs was performed with GOrilla (Gene Ontology enRIchment anaLysis and visuaLizAtion tool, Eden et al., 2009). The Gorilla database is periodically updated using the GO database (Gene Ontology Consortium) and other sources and we used the updated version of December 2016. The* p*-value threshold was equal to 1x10^−3^ and an FDR correction was performed on *p*-values. For gene networks, we also performed functional analyses that used QIAGEN’s Ingenuity Pathway Analysis (IPA®, QIAGEN, Redwood City, CA, USA, http://www.qiagen.com/ingenuity, release date 2016–12-05) with default parameters. eQTL- and ASE-SNPs were annotated using the Variant Effect Predictor [21] to determine their known effects on gene, transcript, and protein sequences.

### Visualization of results

To visualize eGWAS results, we used Cytoscape v3.2.1 [24], which enables the construction of association networks. We used genes and eQTL-SNPs as nodes and significant associations as edges. The R package RCirco [53] was used to illustrate master regulators, which we defined as SNPs that had associations with multiple genes. To generate Venn diagrams, we used jvenn, a plug-in for the jQuery Javascript library [54]. Boxplots were produced with the R package ggplot. The R package PCIT [25] was used to analyze the correlation between the expression variation of probes associated with the same eQTL-SNP.

## Additional files

Additional file 1: Table S1. Associations detected by eGWAS. (XLSX 342 kb)

Additional file 2: Table S2. Genes annotated by sense and/or antisense probes found associated with local or distant eQTL-SNPs. (DOCX 11 kb)

Additional file 3: Figure S1. Distribution of eQTL-SNPs along pig autosomal chromosomes. The histograms represent the distribution of eQTL-SNPs along chromosomes, with each bar corresponding to a 5-Mb segment. The density of eQTL-SNPs is represented by a color gradient from green to red. The scale for the eQTL-SNP count is on the Y-axis and varies among chromosomes. On SSC7, the region between 20 Mb and 30 Mb that has a high density of eQTL-SNPs overlaps the MHC locus. (PDF 30 kb)

Additional file 4: Table S3. ASE analysis results. (XLSX 126 kb)

Additional file 5: Figure S2. Distribution of ASE-SNPs along pig autosomal chromosomes. The histograms represent the distribution of ASE-SNPs along chromosomes, with each bar corresponding to a 5-Mb segment. The density of ASE-SNPs is represented by a color gradient from green to red. The scale for the eQTL-SNP count is on the Y-axis and among chromosomes. The highest density of ASE-SNPs was found on SSC7 at the MHC locus. (PDF 29 kb)

Additional file 6: Table S4. Enrichement analysis of associated genes in eGWAS by GOrilla. (XLSX 39 kb)

Additional file 7: Table S5. Enrichement analysis of local-associated genes in eGWAS by GOrilla. (XLSX 28 kb)

Additional file 8: Table S6. Enrichement analysis of distant-associated genes in eGWAS by GOrilla. (XLSX 21 kb)

Additional file 9: Table S7. Enrichement analysis of cis-genes in ASE analysis by Gorilla. (XLSX 67 kb)

Additional file 10: Figure S3. Comparison of enriched functions for genes detected by eGWAS (total, local, distant), and ASE analysis. A: Venn diagram produced with jvenn [54] showing shared and specific gene ontology (GO) terms. The numbers of enriched GO terms are shown for all associated genes detected by eGWAS (green), distant- (blue) or local-associated genes (red) detected by eGWAS, and ASE-genes (yellow). B: Number of enriched GO terms detected for each analysis. (PNG 40 kb)

Additional file 11: Figure S4. Gene association network centered on eQTL-SNP H3GA0029721. A: Circo plot produced by R package RCirco which maps eQTL-SNP H3GA0029721 and its associated genes according to their genomic position. The blue links represent associations between this eQTL-SNP on SSC10 and 47 genes that map onto other chromosomes. B: The distribution of all (grey) and significant (red) correlation coefficients calculated from pairwise comparisons of expression variation in probes associated with H3GA0029721, performed by the PCIT algorithm. (PDF 131 kb)

Additional file 12: Figure S5. Detailed analysis of the association network centered on eQTL-SNP MARC0001946. A: Circo plot produced by R package RCirco which maps eQTL-SNP MARC0001946 and its associated genes according to their genomic positions. Red links represent associations between MARC0001946 and genes on SSC3, and blue links represent associations with genes on other chromosomes. B: The boxplots represent the expression variation of probes annotated for the porcine genes *UROC1* and *ARHGAP2*, depending on eQTL-SNP genotype. Each dot represents one animal. Pink dots correspond to pigs homozygous for the more-frequent allele (genotype A/A), green dots to heterozygous pigs (genotype A/G), and blue dots to animals homozygous for the less-frequent allele (G/G). The boxplots show that the eQTL-SNP genotypes affected the transcription level of the two associated genes in opposite directions. C: The distribution of all (grey) and significant (red) correlation coefficients calculated from pairwise comparisons of expression variation in probes associated with MARC0001946, performed by the PCIT algorithm. D: Three functional networks produced by IPA, on which genes associated with eQTL-SNP MARC0001946 are represented by gray symbols. The functional network related to “cellular development”, “hematological system development and function”, and “hematopoiesis” includes ten associated genes; the network related to “inflammatory response”, “cell signaling”, and “molecular transport” includes nine associated genes; and the network related to “developmental disorder”, “hereditary disorder”, and “organismal injury and abnormalities” includes five associated genes. (PDF 608 kb)

Additional file 13: Figure S6. Detailed analysis of the association networks centered around eQTL-SNPs ALGA0110383 and MARC0056482. A: Circo plot produced by R package RCirco which maps the two eQTL-SNPs on SSC6 together with their associated genes, according to their genomic positions. Green links represent associations between genes and ALGA0110383. Purple links represent associations between genes and MARC0056482. B: The distribution of all (grey) and significant (red) correlation coefficients calculated from pairwise comparisons of expression variation in probes associated with ALGA0110383 and MARC0001946, performed by the PCIT algorithm. C: Functional network produced by IPA in which genes associated with eQTL-SNPs ALGA0110383 and MARC0001946, as well as the genes *YIPF1* and *PCSK9* (which harbor an ASE-SNP and an eQTL-SNP, respectively) are represented by gray-colored symbols. The functional network, which contains 14 associated genes, is related to “cell death and survival”, “organismal injury and abnormalities”, and “tissue morphology”. D: The boxplots represent expression variations in probes annotated for porcine genes according to each eQTL-SNP genotype. Each dot represents an animal. Pink dots correspond to pigs homozygous for the most-frequent allele (A/A), green dots to heterozygous pigs (A/G), and blue dots to animals homozygous for the less-frequent allele (G/G). (PDF 594 kb)

Additional file 14: Table S8. QTLs in AnimalQTLdb (<10 Mb) for pigs harbouring at least one eQTL-SNP. (XLSX 90 kb)

Additional file 15: Table S9. QTLs in AnimalQTLdb for pigs (<10 Mb) harbouring at least one ASE-SNPs. (XLSX 44 kb)

Additional file 16: Table S10. Complete Blood Cell Counts for 195 animals among the 243 eGWAS pigs. (XLSX 14 kb)

## Abbreviations

ASE: Allele-specific expression
eGWAS: expression genome-wide association study
eQTL: expression quantitative trait locus
FASTA: Family bAsed Score Test for Associations
GO: Gene ontology
GWAS: Genome-wide association study
IPA: Ingenuity pathway analysis
MHC: Major Histocompatibility Complex
PCIT: Partial correlation information theory
QC: Quality control
QTL: Quantitative trait locus
SLA: Swine Leucocyte Antigen
SNP: Single nucleotide polymorphism
VEP: Variant effect predictor
