# Supplementary material for: Deciphering the genetic regulation of peripheral blood transcriptome in pigs through expression genome-wide association study and allele-specific expression analysis
Source: BMC Genomics. 2017 Dec 13;18:967. doi: 10.1186/s12864-017-4354-6 (PMC5729405; doi:10.1186/s12864-017-4354-6)
Supplement: Supplementary file 2 — Genes annotated by sense and/or antisense probes found associated with local or distant eQTL-SNPs. (DOCX 11 kb) [file 12864_2017_4354_MOESM2_ESM.docx]

**Additional file 2: Table S2. Genes annotated by sense and/or antisense probes found associated with local or distant eQTL-SNPs**

|  | **Local** | **Distant** | **Local+Distant** | **Total** |
| --- | --- | --- | --- | --- |
| Genes annotated with sense-probes | 990 | 526 | 312 | 1828 |
| Genes annotated with sense and antisense probes | 29 | 5 | 39 | 73 |
| **Total** | **1019** | **531** | **351** | **1901** |
| Genes annotated with antisense-probes | 115 | 108 | 21 | 244 |
| Non-annotated probes | 220 | 147 | 54 | 421 |
