# Supplementary figures and images for: Deciphering the genetic regulation of peripheral blood transcriptome in pigs through expression genome-wide association study and allele-specific expression analysis
Source: BMC Genomics. 2017 Dec 13;18:967. doi: 10.1186/s12864-017-4354-6 (PMC5729405; doi:10.1186/s12864-017-4354-6)

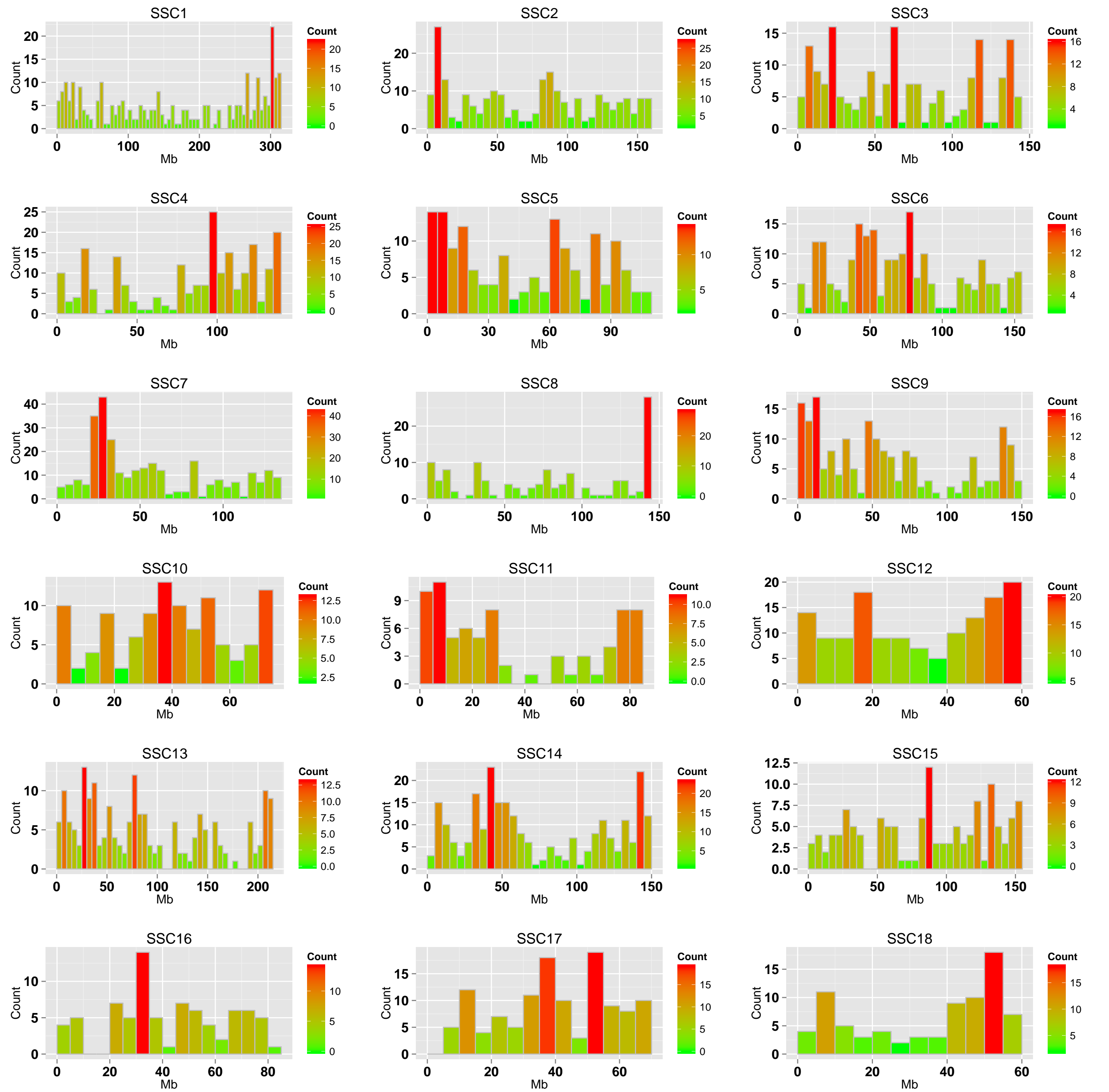

Supplement: Supplementary file 3 — Distribution of eQTL-SNPs along pig autosomal chromosomes. The histograms represent the distribution of eQTL-SNPs along chromosomes, with each bar corresponding to a 5-Mb segment. The density of eQTL-SNPs is represented by a color gradient from green to red. The scale for the eQTL-SNP count is on the Y-axis and varies among chromosomes. On SSC7, the region between 20 Mb and 30 Mb that has a high density of eQTL-SNPs overlaps the MHC locus. (PDF 30 kb) [file 12864_2017_4354_MOESM3_ESM.pdf]

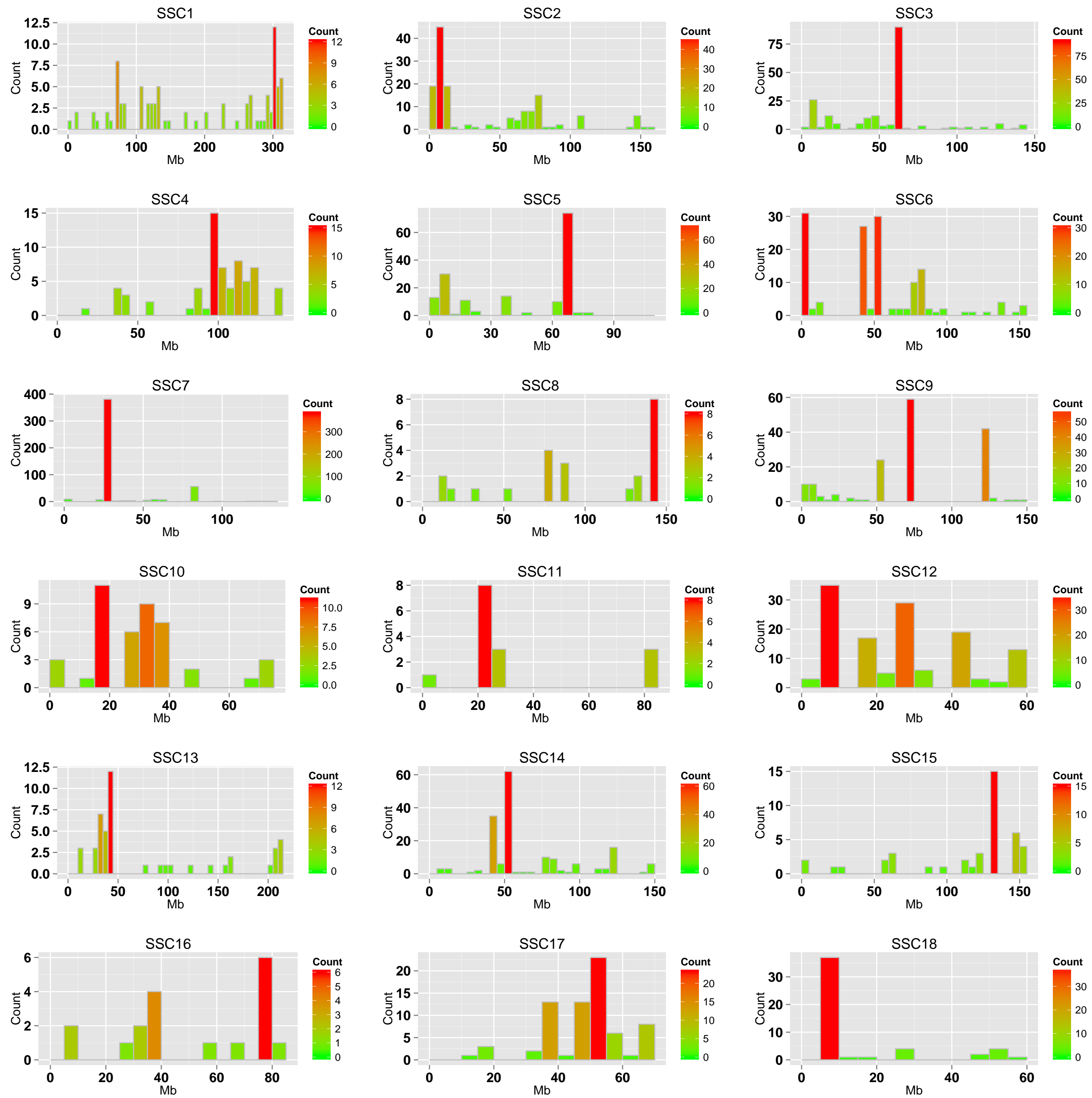

Supplement: Supplementary file 5 — Distribution of ASE-SNPs along pig autosomal chromosomes. The histograms represent the distribution of ASE-SNPs along chromosomes, with each bar corresponding to a 5-Mb segment. The density of ASE-SNPs is represented by a color gradient from green to red. The scale for the eQTL-SNP count is on the Y-axis and among chromosomes. The highest density of ASE-SNPs was found on SSC7 at the MHC locus. (PDF 29 kb) [file 12864_2017_4354_MOESM5_ESM.pdf]

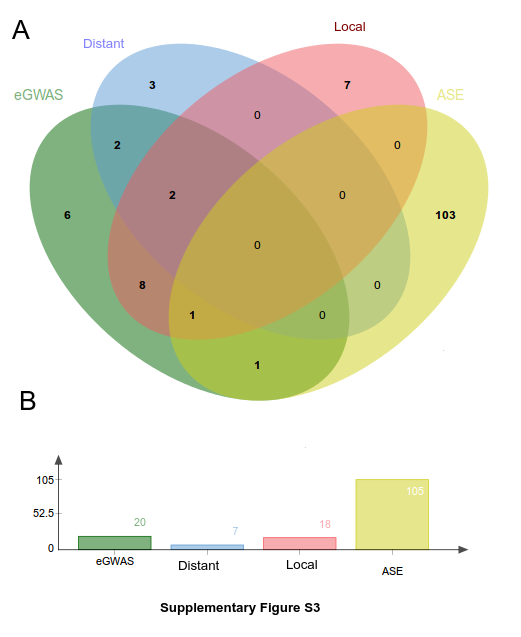

Supplement: Supplementary file 10 — Comparison of enriched functions for genes detected by eGWAS (total, local, distant), and ASE analysis. A: Venn diagram produced with jvenn [54] showing shared and specific gene ontology (GO) terms. The numbers of enriched GO terms are shown for all associated genes detected by eGWAS (green), distant- (blue) or local-associated genes (red) detected by eGWAS, and ASE-genes (yellow). B: Number of enriched GO terms detected for each analysis. (PNG 40 kb) [file 12864_2017_4354_MOESM10_ESM.png]

A

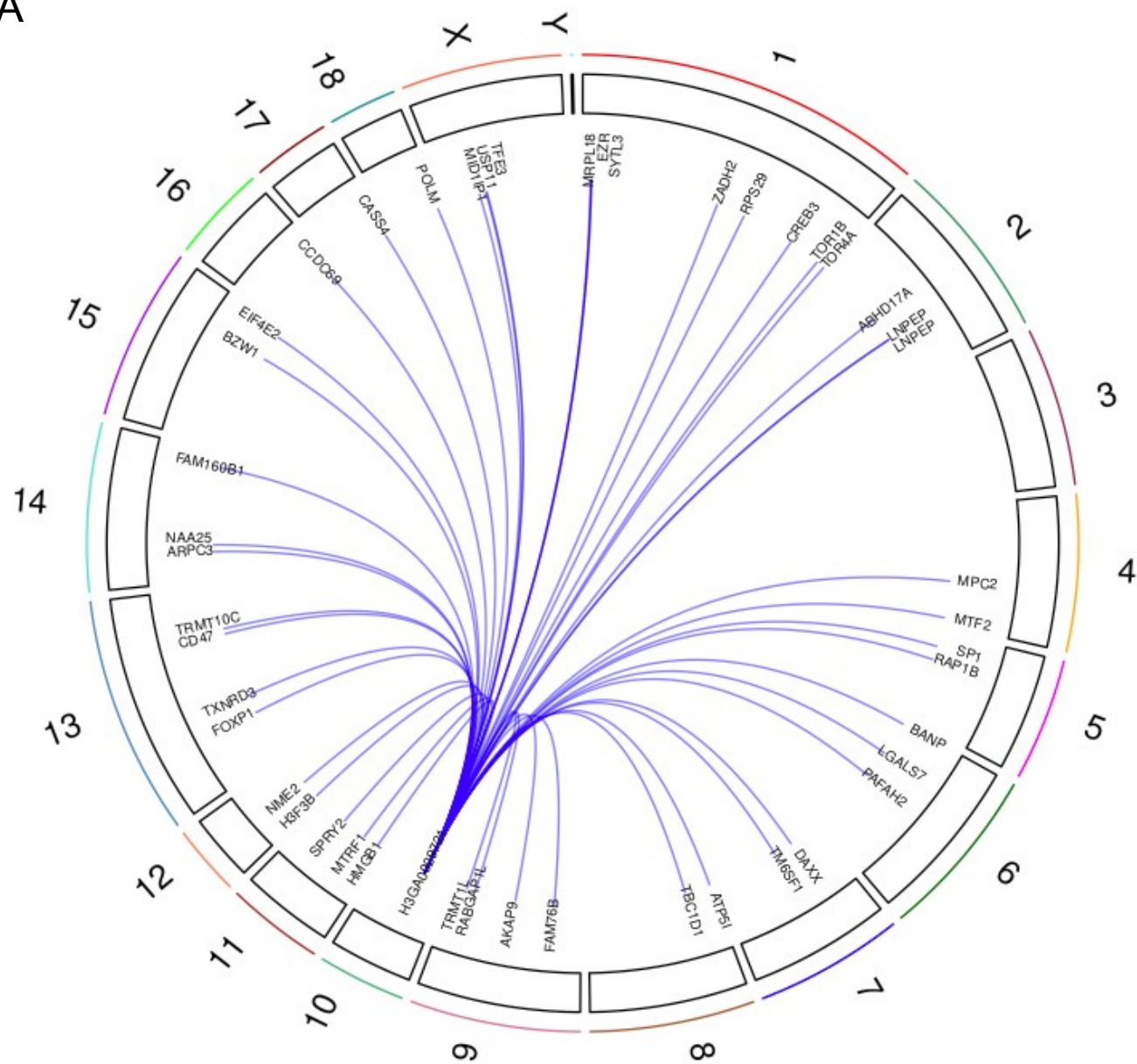

**B**

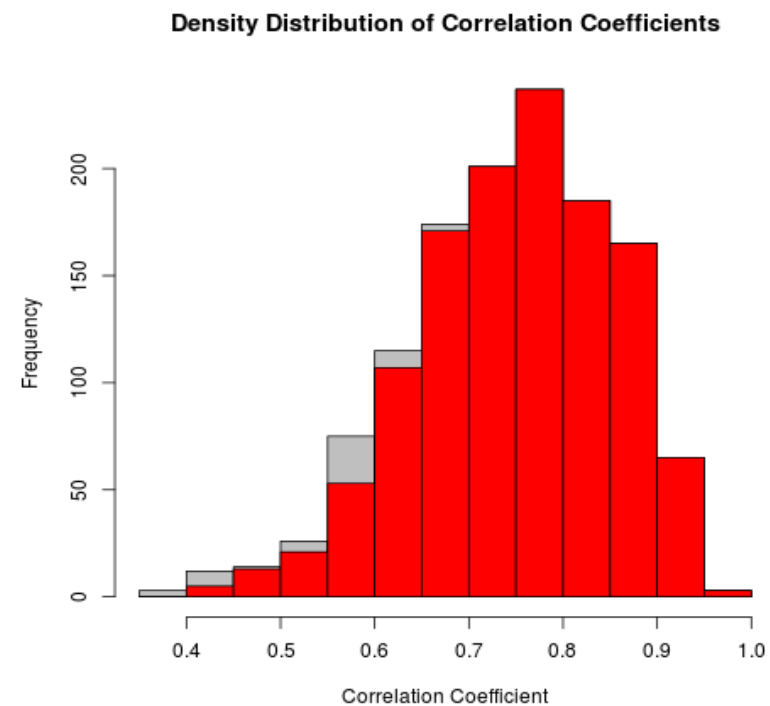

### Supplementary Figure S4

Supplement: Supplementary file 11 — Gene association network centered on eQTL-SNP H3GA0029721. A: Circo plot produced by R package RCirco which maps eQTL-SNP H3GA0029721 and its associated genes according to their genomic position. The blue links represent associations between this eQTL-SNP on SSC10 and 47 genes that map onto other chromosomes. B: The distribution of all (grey) and significant (red) correlation coefficients calculated from pairwise comparisons of expression variation in probes associated with H3GA0029721, performed by the PCIT algorithm. (PDF 131 kb) [file 12864_2017_4354_MOESM11_ESM.pdf]

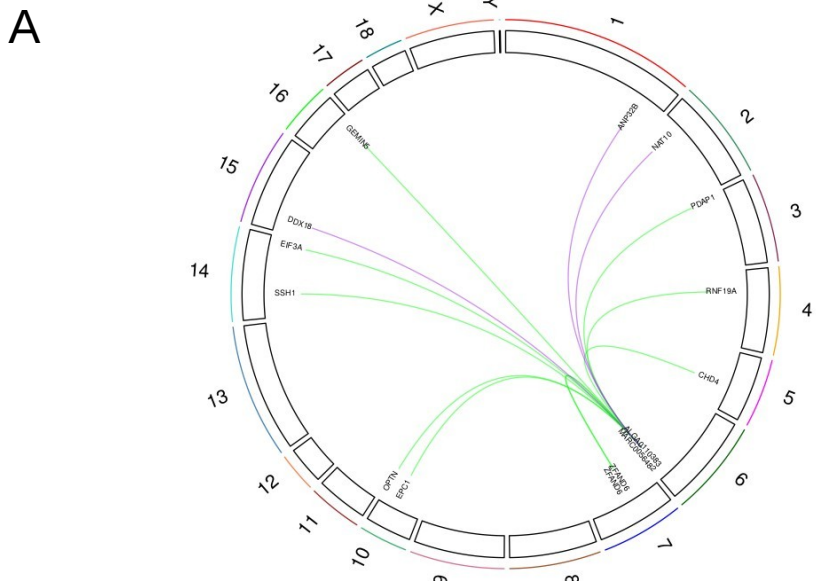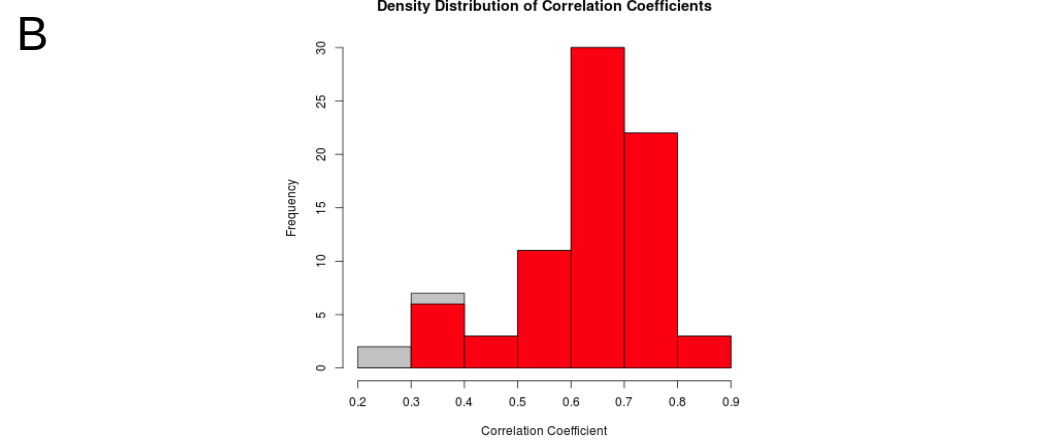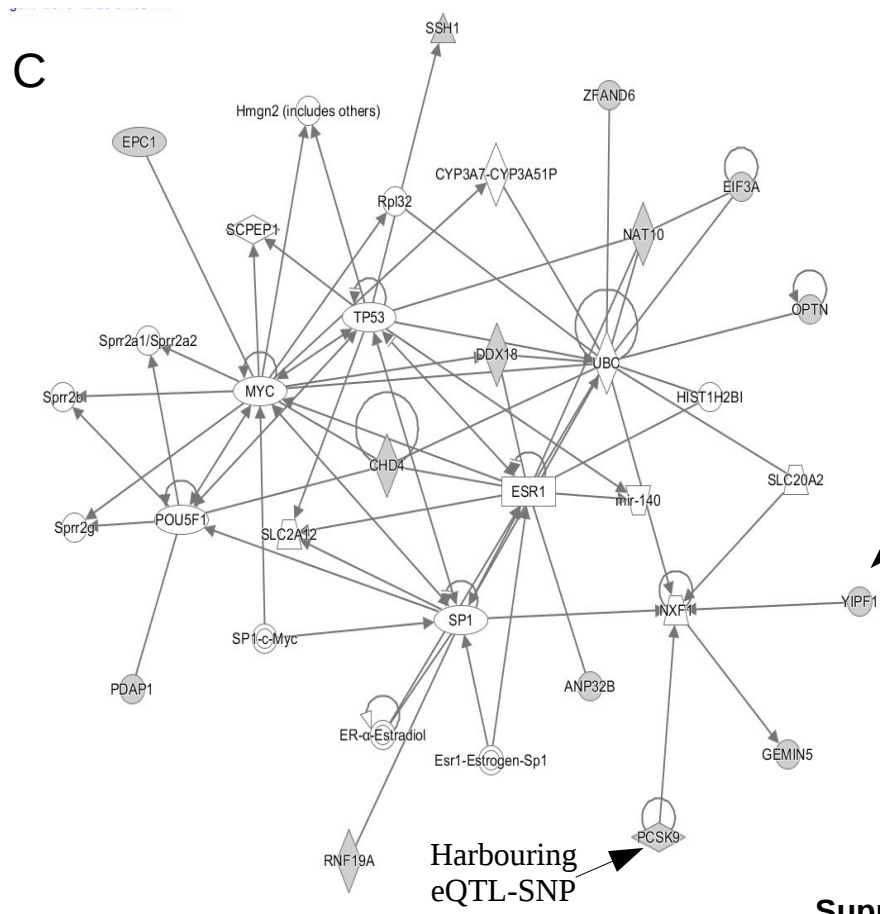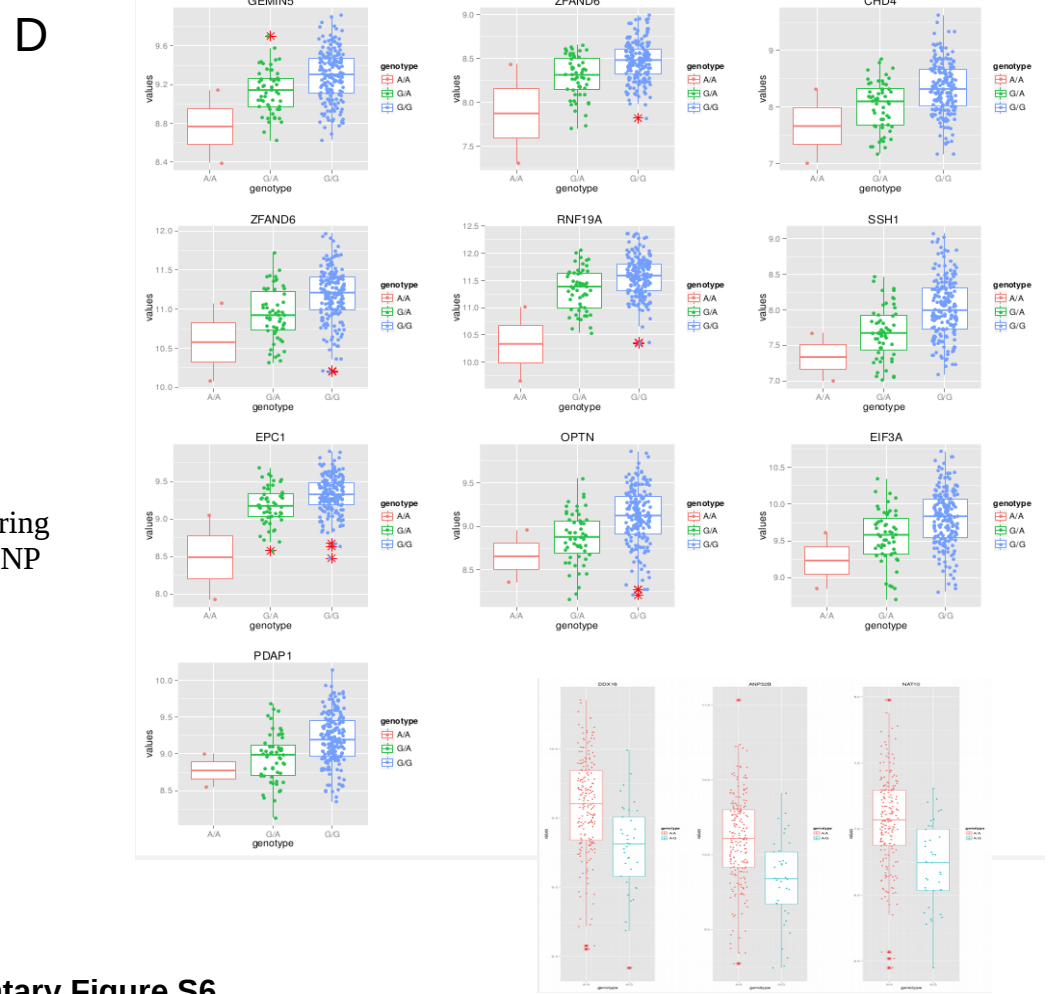

Supplementary Figure S6

Supplement: Supplementary file 13 — Detailed analysis of the association networks centered around eQTL-SNPs ALGA0110383 and MARC0056482. A: Circo plot produced by R package RCirco which maps the two eQTL-SNPs on SSC6 together with their associated genes, according to their genomic positions. Green links represent associations between genes and ALGA0110383. Purple links represent associations between genes and MARC0056482. B: The distribution of all (grey) and significant (red) correlation coefficients calculated from pairwise comparisons of expression variation in probes associated with ALGA0110383 and MARC0001946, performed by the PCIT algorithm. C: Functional network produced by IPA in which genes associated with eQTL-SNPs ALGA0110383 and MARC0001946, as well as the genes YIPF1 and PCSK9 (which harbor an ASE-SNP and an eQTL-SNP, respectively) are represented by gray-colored symbols. The functional network, which contains 14 associated genes, is related to “cell death and survival”, “organismal injury and abnormalities”, and “tissue morphology”. D: The boxplots represent expression variations in probes annotated for porcine genes according to each eQTL-SNP genotype. Each dot represents an animal. Pink dots correspond to pigs homozygous for the most-frequent allele (A/A), green dots to heterozygous pigs (A/G), and blue dots to animals homozygous for the less-frequent allele (G/G). (PDF 594 kb) [file 12864_2017_4354_MOESM13_ESM.pdf]
