# Supplementary material for: Deciphering the genetic regulation of peripheral blood transcriptome in pigs through expression genome-wide association study and allele-specific expression analysis
Source: BMC Genomics. 2017 Dec 13;18:967. doi: 10.1186/s12864-017-4354-6 (PMC5729405; doi:10.1186/s12864-017-4354-6)
Supplement: Supplementary file 12 — Detailed analysis of the association network centered on eQTL-SNP MARC0001946. A: Circo plot produced by R package RCirco which maps eQTL-SNP MARC0001946 and its associated genes according to their genomic positions. Red links represent associations between MARC0001946 and genes on SSC3, and blue links represent associations with genes on other chromosomes. B: The boxplots represent the expression variation of probes annotated for the porcine genes UROC1 and ARHGAP2, depending on eQTL-SNP genotype. Each dot represents one animal. Pink dots correspond to pigs homozygous for the more-frequent allele (genotype A/A), green dots to heterozygous pigs (genotype A/G), and blue dots to animals homozygous for the less-frequent allele (G/G). The boxplots show that the eQTL-SNP genotypes affected the transcription level of the two associated genes in opposite directions. C: The distribution of all (grey) and significant (red) correlation coefficients calculated from pairwise comparisons of expression variation in probes associated with MARC0001946, performed by the PCIT algorithm. D: Three functional networks produced by IPA, on which genes associated with eQTL-SNP MARC0001946 are represented by gray symbols. The functional network related to “cellular development”, “hematological system development and function”, and “hematopoiesis” includes ten associated genes; the network related to “inflammatory response”, “cell signaling”, and “molecular transport” includes nine associated genes; and the network related to “developmental disorder”, “hereditary disorder”, and “organismal injury and abnormalities” includes five associated genes. (PDF 608 kb) [file 12864_2017_4354_MOESM12_ESM.pdf]

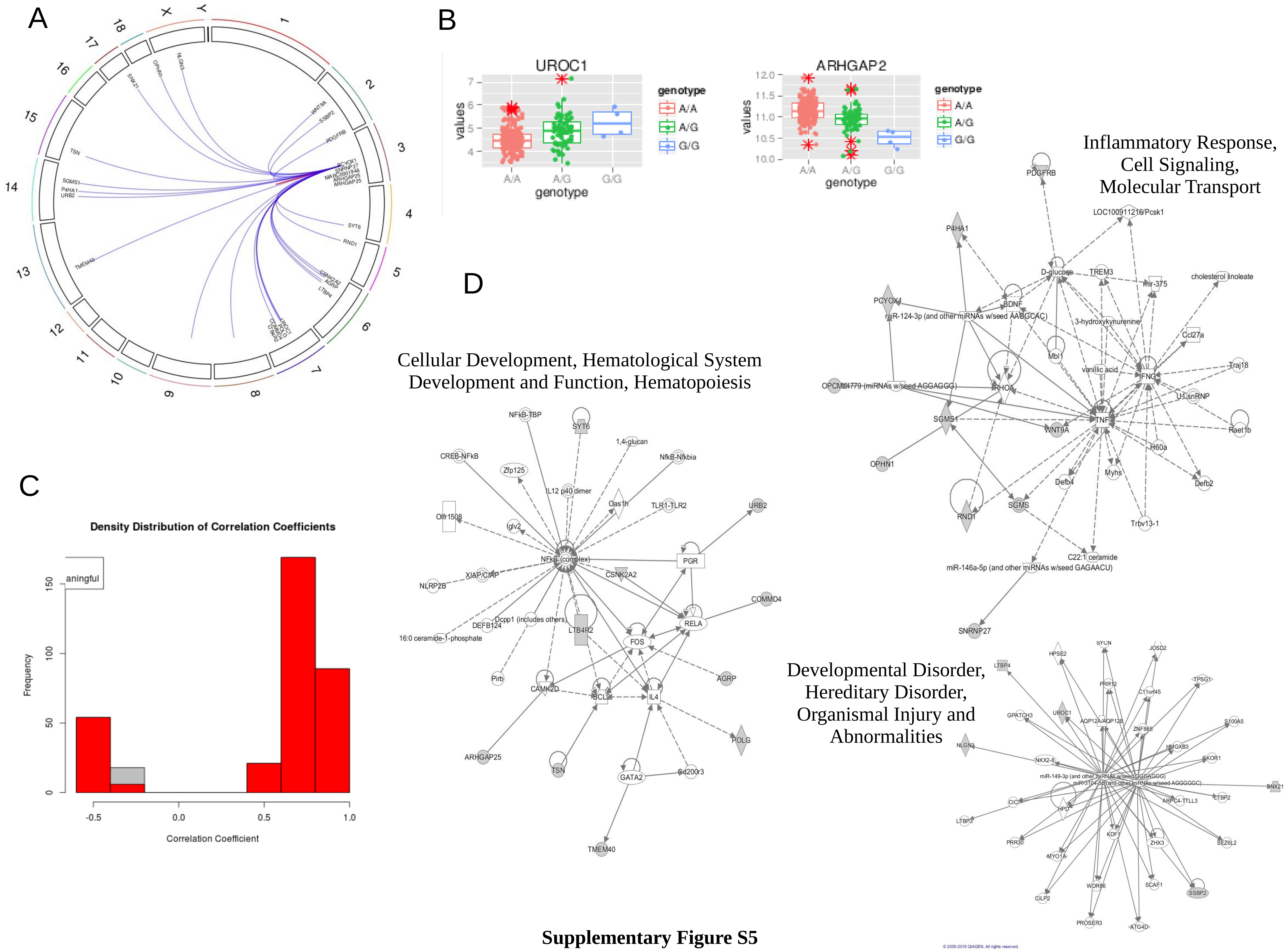

Supplementary Figure S5
